# The factors affecting neurogenesis after stroke and the role of acupuncture

**DOI:** 10.3389/fneur.2023.1082625

**Published:** 2023-01-20

**Authors:** Jie-Dan Mu, Liang-Xiao Ma, Zhou Zhang, Xu Qian, Qin-Yong Zhang, Ling-Hui Ma, Tian-Yi Sun

**Affiliations:** ^1^School of Acupuncture-Moxibustion and Tuina, Beijing University of Chinese Medicine, Beijing, China; ^2^The Key Unit of State Administration of Traditional Chines Medicine, Evaluation of Characteristic Acupuncture Therapy, Beijing, China

**Keywords:** stroke, neurogenesis, axon growth, NGF, Rho A, slit, acupuncture

## Abstract

Stroke induces a state of neuroplasticity in the central nervous system, which can lead to neurogenesis phenomena such as axonal growth and synapse formation, thus affecting stroke outcomes. The brain has a limited ability to repair ischemic damage and requires a favorable microenvironment. Acupuncture is considered a feasible and effective neural regulation strategy to improve functional recovery following stroke *via* the benign modulation of neuroplasticity. Therefore, we summarized the current research progress on the key factors and signaling pathways affecting neurogenesis, and we also briefly reviewed the research progress of acupuncture to improve functional recovery after stroke by promoting neurogenesis. This study aims to provide new therapeutic perspectives and strategies for the recovery of motor function after stroke based on neurogenesis.

## 1. Introduction

Despite considerable efforts over the last decades, stroke remains the leading cause of death and disability worldwide, placing a severe economic burden on countries (1, 2). Functional recovery following a stroke is exceptionally limited, leaving the affected individual with life-long neurological deficits (3). This lack of functional recovery can at least in part be attributed to the restriction of neurogenesis (4, 5).

Neuroplasticity is a native ability of the brain to adapt to individual developmental growth. The historical view of the central nervous system (CNS) as a static organ has shifted in recent years (6). We now realized that CNS remains plastic and has some regeneration capacity to rebuild neural circuits after acute injuries, such as stroke (6, 7). Stroke induces a state of neuroplasticity. This period of enhanced plasticity provides an opportunity for neurogenesis, such as the sprouting of new axons, the formation of new synapses, and the remapping of sensorimotor functions, which is associated with motor recovery (8). The reconstruction of neural circuits is generated in the sensorimotor cortex, thalamus, brain stem, and spinal cord (9, 10).

The compensatory repair capacity of the brain for ischemic injury is limited and a favorable microenvironment is needed (11). After a stroke, the reconstruction of neural circuits is restricted by the presence of many inhibitory factors that inhibit neurogenesis in the local microenvironment, the lack of growth factors, as well as the formation of glial scars in the injured area (12). An enriched microenvironment exerts a significant influence on neurogenesis (13, 14). As such, improving the microenvironment of the injured site, thus promoting neurogenesis and the reconstruction of neural circuits, has been the focus of intense research.

Acupuncture is a form of physical stimulation therapy that originated in traditional Chinese medicine. Needles are inserted into the skin or deep tissues at specific locations (acupoints) on the body, and stimulation is enhanced by specific needling techniques or by electricity to restore body balance, prevent, and treat disease (15). Modern neuroanatomical evidence has demonstrated that there are abundant nerve endings in the acupuncture meridian and acupoint areas of the body, while the achievement of therapeutic effects of acupuncture mainly depends on the nervous system (16, 17). A growing number of clinical studies have shown that acupuncture can effectively improve recovery from stroke (18, 19). The mechanism of acupuncture effects may be related to neuroplasticity.

Here, we summarized our current understanding of the key factors and signaling pathways that affect CNS neurogenesis. Meanwhile, we briefly overviewed the research progress of acupuncture in improving motor function after stroke by accelerating neurogenesis. We hope to emphasize that neurogenesis could be modulated by potential strategies to improve functional outcomes after stroke and explore the feasibility of acupuncture in promoting motor function recovery after stroke. The aim is to provide new therapeutic opportunities for post-stroke motor rehabilitation based on neurogenesis.

## 2. Enhancing factors of neurogenesis

Neurogenesis is the process through which neural stem cells (NSCs), or more generally neural progenitor cells (NPCs), generate new neurons (20). The adult CNS contains NSCs that can continuously generate neurons, astrocytes, and oligodendrocytes (21, 22). Under normal conditions, the NPCs are in a quiescent state in the adult brain. After an ischemic injury, NPCs proliferate, differentiate, and migrate to the ischemic region to replenish neurons in the damaged area, release the anti-inflammatory cytokines to limit the deleterious inflammatory environment, and form the new neuronal connections to promote the recovery of nerve function and resist ischemic injury (23–25). Neurogenesis in the brain of adult mammals has been clearly demonstrated that mainly occurs in the subventricular zone (SVZ) of the lateral ventricle and the subgranular zone (SGZ) of the dentate gyrus in the hippocampus (21, 22, 26). After the cerebral ischemic injury, multiple growth factors and a variety of proteins are increasingly expressed to promote the proliferation of NSCs or NPCs, and thus facilitate neurogenesis, which includes the production of new neurons, glia, axons, myelin sheaths, or synapses (6, 22, 27).

### 2.1. Growth factors

**Figure 1:**
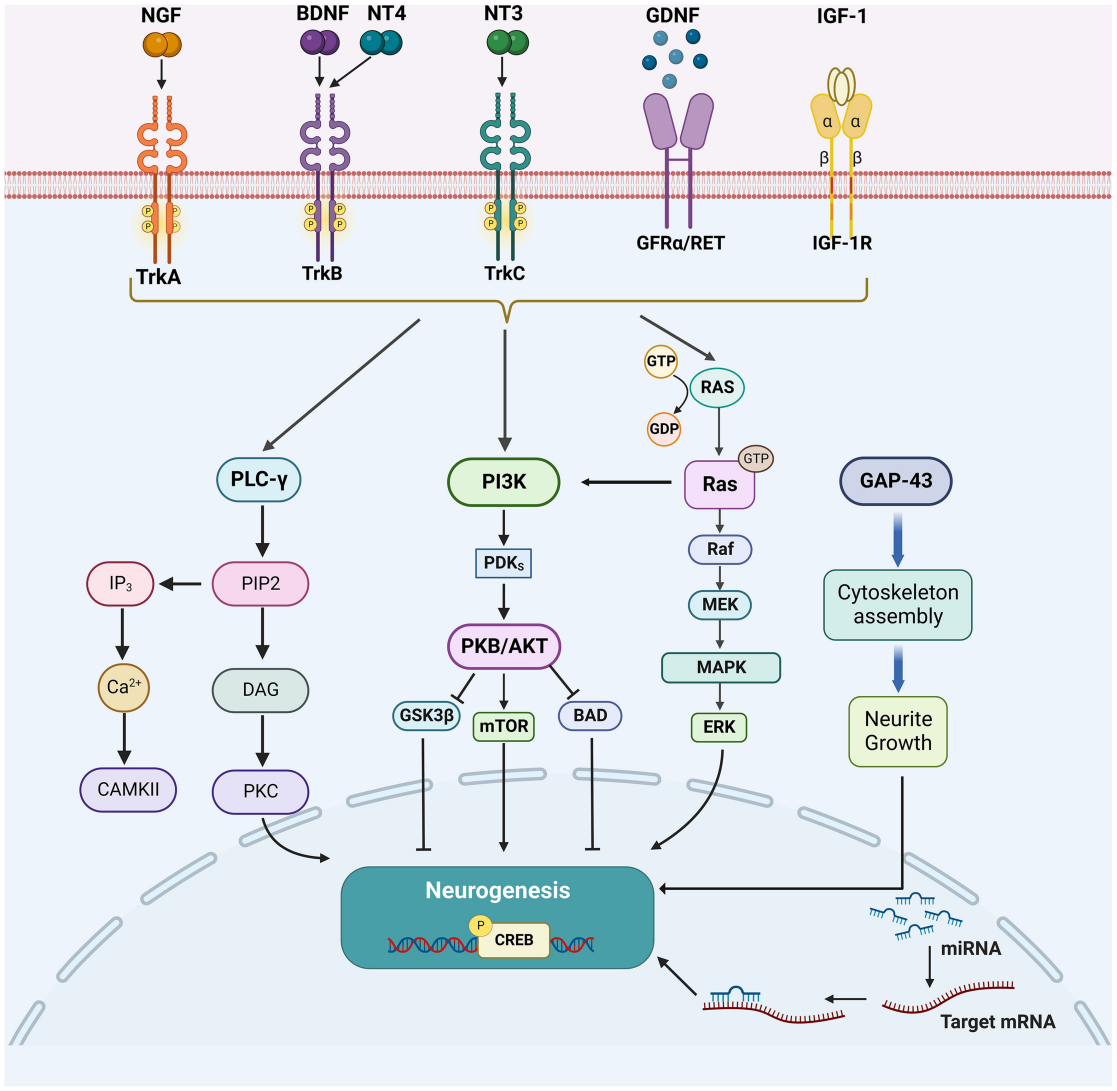
The enhancing factors of neurogenesis and their major downstream signaling pathways. After the cerebral ischemic injury, multiple growth factors (NGF, BDNF, NT3/4, and GDNF), other growth proteins (GAP-43 and IGF-1), and miRNAs could promote neurogenesis. Their downstream signaling cascades, mainly include PLC-γ/PKC, PI3K/Akt, MAPK/ERK, and Wnt/β-catenin pathways.

Growth factors, such as nerve growth factor (NGF), brain-derived neurotrophic factor (BDNF), glial cell lineage-derived neurotrophic factor (GDNF), neurotrophin-3/4 (NT-3/4), and insulin-like growth factor 1 (IGF-1) play an important role in neuronal survival, differentiation as well as axonal regeneration of damaged CNS neurons (28–30). Their downstream signaling cascades, mainly including phospholipase C-γ (PLC-γ), phosphatidylinositol 3-kinase (PI3K), and mitogen-activated protein kinase (MAPK) pathways, have also been involved in promoting neurogenesis after stroke (31). The activation of MAPK/extracellular signal-regulated kinase (ERK) pathway starts from RAS. The activated RAS protein recruits downstream RAF proteins located in the cytoplasm and further activates downstream MEK through its CR3 structural domain at the C-terminus, which in turn activates MAPK and ERK. ERK phosphorylates and activates the downstream transcription factor CREB to regulate the expression of target genes that contribute to neuronal differentiation and survival (32). PI3K plays an important role in regulating cell survival, axon growth, and cytoskeleton remodeling by recruiting protein kinase B (Akt) to activate mammalian targets of rapamycin (mTOR), and phosphorylation and inhibiting apoptosis-promoting proteins such as Bad and GSK3β (33, 34). PLC-γ is activated to convert extracellular stimuli into intracellular signals by hydrolyzing phosphatidylinositol 4,5-bisphosphate (PIP2) to generate second messengers inositol triphosphate (IP3) and diacylglycerol (DAG). DAG activates PKC, while IP3 induces calcium release from intracellular calcium depots (35, 36) (Figure 1).

#### 2.1.1. NGF

NGF is present in tissues mainly in the form of precursors and promotes the survival and differentiation of neurons in the nervous system (37). TrkA is a specific receptor for NGF (38). Activation of TrkA leads to the phosphorylation of tyrosine residues in its structural domain, which recruits signaling molecules and activates multiple signaling pathways including PLC-γ, MAPK, and PI3K (38, 39). Among them, phosphorylation of Y490 and Y785 is the most common (40). Phosphorylated Y490 recruits Src homologous and collagen (Shc) to activate MAPK and PI3K pathways, while phosphorylated Y785 recruits the PLC-γ1 pathway (41, 42).

It has been demonstrated that NGF can protect sensory neurons and promote neurogenesis in the damaged area after stroke (43). On the one hand, NGF could increase the survival of NPCs through the activation of TrkA, in turn, induces axonal outgrowth (the elaboration of axonal filopodia and branches) and myelination (38, 39). On the other hand, for the survival of newly generated neurons after stroke, the NGF is also involved in angiogenesis after acute stroke in rats (44). It demonstrates that NGF is involved in multiple processes in neurogenesis after stroke.

#### 2.1.2. BDNF

BDNF is the most abundant and most studied neurotrophic factor (NTF) in the brain (29). BDNF can activate three signaling proteins, MAPKs, PI3Ks, and PLC-γ, by interacting with tropomyosin receptor TrkB (45). It has also been found that BDNF promotes neurogenesis by activating the JAK/STAT pathway in Schwann cells (46).

A study has shown that BDNF has a neuroprotective effect on hippocampal neuronal damage caused by hypoxic and glucose-deficient conditions (47). BDNF not only has an important role in promoting neuronal survival but also promotes neurogenesis after ischemic stroke (48). Therefore, modulation of BDNF expression is promising for neurogenesis and protrusion generation in damaged areas after stroke.

#### 2.1.3. Neurotrophic factors

Multiple NTFs act together to promote axonal growth during neurological maturation (49). Among them, NT3 and NT4 have the most potential for neurogenesis after stroke (50). NT3 binds mainly to TrkC receptors specifically and NT4 binds mainly to TrkB specifically (51). NT3 is a neurotrophic factor that plays an important role in preventing the death of damaged neurons, enhancing neuronal survival and axonal regeneration, and inducing the differentiation of endogenous oligodendrocyte precursor cells into mature oligodendrocytes to restore myelin (52). Studies have shown that NT3 can be transported from muscle to sensory ganglia and spinal motor neurons in nerve, as well as to the CNS through the bloodstream (30, 49, 53, 54). NT3 can promote axonal growth and synaptic plasticity in various locomotor pathways including the corticospinal tract and proprioceptive pathways and can induce axonal growth from the intact corticospinal tract across the midline to the innervated side (30, 49). In addition, Ann et al. have demonstrated that NTF synergistic neuronal responses after a combination of basic fibroblast growth factor, NT3 and BDNF delivered to the somata of retinal ganglion cells promoted greater survival and axon growth than did the sum of the effects of each NTF alone (55).

Regarding NT4, it has been shown that stroke mice knocking out the NT4 gene exhibit larger infarct foci, suggesting that NT4 can counteract ischemic brain injury (56). Another study has also shown that post-stroke neurological recovery from exercise is closely related to NT4 (57).

#### 2.1.4. GDNF

Among the many endogenous regulatory molecules, GDNF is particularly notable as it is produced by glial cells and neurons and is a member of the transforming growth factor β superfamily, which plays an important role in neuronal differentiation during normal development (58). GDNF family ligands bind to specific GDNF family receptor alpha (GFRα), all these form receptor complexes and signal through the RET receptor tyrosine kinase and activate downstream PLC-γ, MAPK and PI3K/Akt signaling pathways (59, 60).

GDNF can promote the survival and recovery of several types of mature neurons after CNS injury. The RET receptor induces calcium (Ca^2+^) signaling and regulates neocortical NPCs migration through the PLC-γ binding domain Tyr1015 (60). In one study, a fusion protein, PEP-1-GDNF, was injected intravenously into rats with stroke, and GDNF was found to significantly reduce infarct size, promote proliferation and differentiation of hippocampal dentate gyrus cells, and improve behavioral function (61). Beker et al. (62) demonstrated that GDNF is effective in inducing long-term neural recovery, peri-infarct brain remodeling, and contralateral neuroplasticity. In addition, activation of GDNF pathways may enhance hippocampal neurogenesis and thus promote neuronal survival (63). Therefore, GDNF is an important target for regulating neurogenesis after cerebral ischemia.

#### 2.1.5. Other growth factors

Growth-associated protein-43 (GAP-43) is the main protein of the axonal growth cone that promotes axonal sprouting during the development and regeneration of the nervous system (64, 65). It promotes the accumulation of F-actin in neural protrusions and contributes to the formation of the cytoskeleton (66). It also promotes the release of presynaptic membrane neurotransmitters, cytokinesis, and circulation of synaptic vesicles, promotes synapse formation, stimulates axon outgrowth and extension, inhibits axon necrosis and growth cone retraction, promotes oligodendrocyte and astrocyte differentiation, and thus promotes neurogenesis (64). The axonal sprouting process is accompanied by high expression of GAP-43 after ischemic stroke (67). GAP-43 in neurons is a substrate for caspase-3 (CASP3) (68), a protease involved not only in apoptosis but also in fine-tuning the formation of new synaptic contacts (69). It was found that GAP-43 and CASP3 are involved in the neurogenesis of lesions after ischemic stroke (70).

Insulin-like growth factor-1 (IGF-1) is a growth factor primarily produced by the liver in adults and plays a crucial role in cell proliferation, maturation, and survival (71). In addition, it has important effects on early CNS development and neuronal plasticity (72). In the CNS, IGF-1 exerts its action by binding to its receptor (IGF1R), a membrane-bound glycoprotein composed of two alpha and two β subunits (73, 74). Once IGF-1 binds to IGF1R, the tyrosine kinase structural domain on the β subunits activates the PI3K/Akt1/mTOR and MAPK/ERK pathways to induce their downstream effects (75, 76). Adeno-associated virus (AAV)-mediated IGF-1 overexpression was found to promote long-term functional recovery in mice with focal ischemia by promoting neovascularization and neurogenesis (77). IGF-1 was also found to regulate the survival and migration of bone marrow mesenchymal stem cells in an ischemic environment and improve neurological recovery after ischemic stroke (28). It suggests that IGF-1 may be a safe and potentially effective treatment for a variety of CNS disorders including ischemic stroke.

### 2.2. MicroRNAs

MicroRNAs (miRs) are a family of 20–25 non-coding RNAs that play an important role in the pathogenesis of ischemic stroke and are important factors in the regulation of axonal growth in neurons (78). The main miRs that promote neurogenesis are miR-133b/30b/132/124/146 (79–83), etc. These molecules can affect a variety of biological processes related to post-stroke axonal growth and synaptic function regulation by targeting hundreds of proteins in a range of cellular and molecular targets and multiple regulatory networks.

Different miRNAs regulate neurogenesis differently. For example, miR-133b promotes neurogenesis by activating MAPK/ERK1/2 and PI3K/Akt signaling pathways (79). The miR-146 family (miR-146a and miR-146b) could promote the differentiation of NSCs into neurons by regulating the Notch1 signaling pathway (83). MiR-30b promotes axon outgrowth of retinal ganglion cells by inhibiting Sema3A-mediated caspase-3 and p38MAPK signaling pathways (80). In addition, miR-124 can activate the Wnt/ß-catenin pathway by targeting DACT1 to promote NSCs proliferation and differentiation to neurons (82). It has also been shown that miR-124 can reduce glial scar formation in M2 microglia and promote neurogenesis in mice after stroke through STAT3 signaling (84). Therefore, miRs may serve as innovative gene therapy candidates for neurogenesis (85).

## 3. Inhibiting factors of neurogenesis

The limited ability to neurogenesis after cerebral ischemia is mainly due to ischemic injury-producing factors that inhibit neurogenesis. The microenvironment within the nervous system is critical for the survival and regeneration of damaged nerves. To some extent, inhibitory factors are thought to possibly play a more important role.

Locally to the injury, the formation of hard glial scarring by glial cells prevents nascent axons from crossing (86). Most of these inhibitory molecules induce the activation of RhoA/ROCK pathway in neurons (87). RhoA/ROCK is one of the most widely studied signaling pathways and is primarily responsible for regulating cytoskeleton organization, cell growth, cell migration, proliferation, and development (87). Inhibition of this pathway can promote axon growth and behavioral recovery in rats after stroke (87, 88).

### 3.1. Myelin proteins

**Figure 2:**
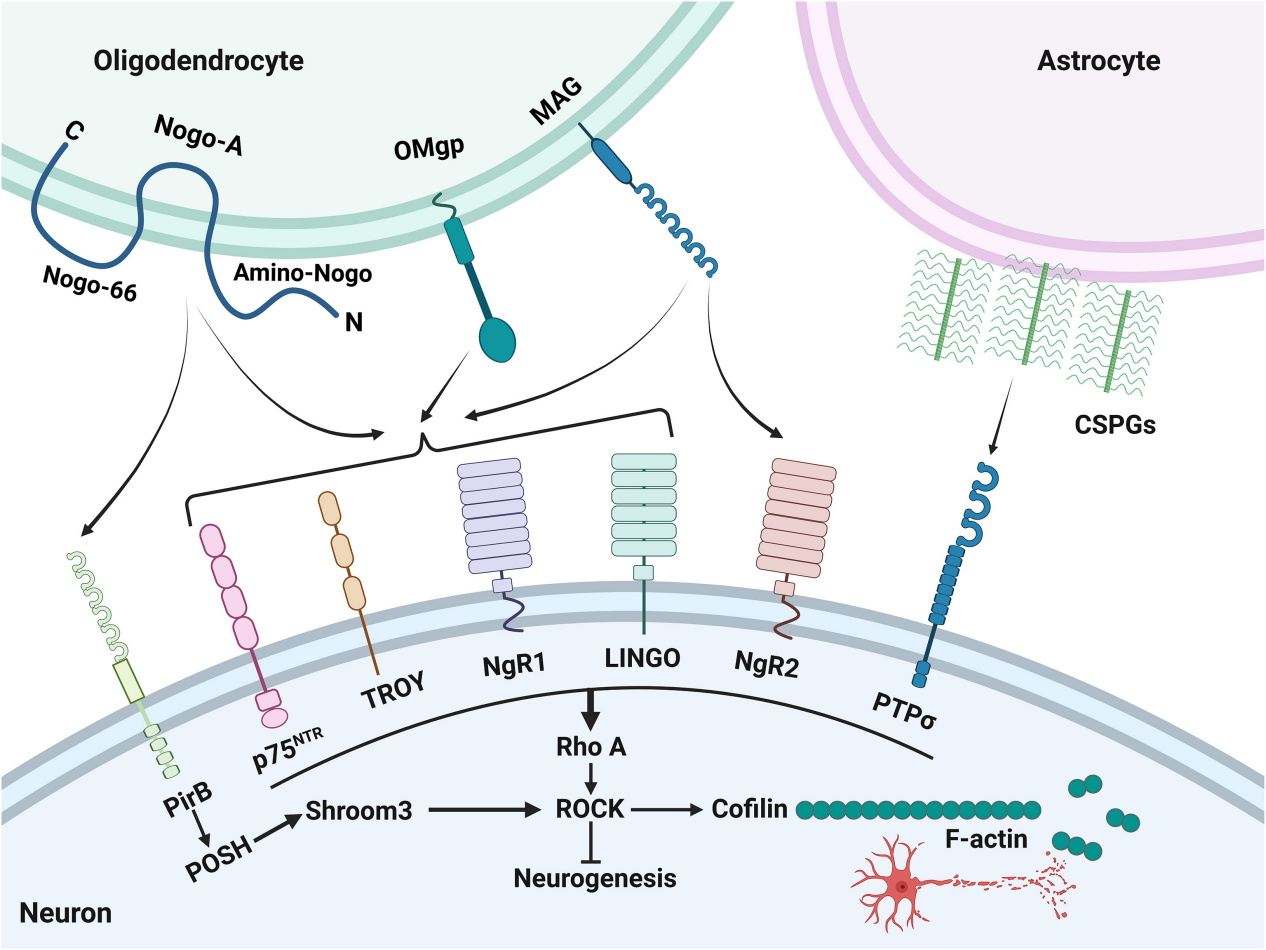
Inhibiting factors of neurogenesis and their major downstream signaling pathways. After an ischemic stroke, various growth inhibitory molecules (Nogo-A, MAG, OMgp, and CSPG) from myelin and reactive astrocytes interact with various receptors, such as PirB, NgR1/2, LINGO, p75^NTR^, PTPσ, and TROY, to signal through a convergent downstream pathway within the axon, leading to collapse of growth cones and inhibition of axonal growth.

There is a consensus that myelin is a major barrier to inhibiting neurogenesis (87). The myelin sheath of PNS appears to be removed faster and more effectively than that of CNS, mainly due to differences in the surrounding microenvironment (89, 90). The myelin sheath of PNS is Schwann cell, which can produce more neurotrophic factors and promote the growth of PNS axons (91). However, the myelin sheath of CNS is oligodendrocyte, which produces more neural growth inhibitory factors and is not conducive to the regeneration of CNS axons (92) (Figure 2). Some factors that inhibit axonal growth such as Nogo, myelin-associated glycoprotein (MAG), and oligodendrocyte-myelin glycoprotein (OMgp) are mainly expressed in CNS myelin (93).

#### 3.1.1. Nogo-A

NOGO-protein family Nogo, especially Nogo-A plays a key role in CNS neurogenesis. Nogo-A and its receptors are widely present in the CNS of mammalian, and they are strongly associated with axonal growth inhibition and neuronal damage caused by ischemic brain injury (94, 95). It was found that spontaneous axonal plasticity and functional recovery after stroke may be limited by Nogo-A (96). Studies have shown that Nogo-A is expressed in several regions of the nervous system to varying degrees after cerebral ischemic injury, and it can inhibit the structural and functional recovery of the corticospinal tract to a certain extent, while the recovery of neurological function can be effectively promoted by antagonizing Nogo-A (95, 97–99).

One of the mechanisms by which Nogo-A exerts these effects is that Nogo-A binds to its receptor (NgR) complex. Nogo-66 stimulates the receptor complex composed of NgR1 and its related proteins LINGO and p75 neurotrophin receptor (p75^NTR^) or TROY to activate the downstream Rho A/ROCK actin disruptor cofilin (a major effector of growth cone cytoskeleton disassembly) signaling pathway, preventing actin cytoskeleton aggregation in the growth cone, eventually leading to collapse of growth cones and inhibition of axonal growth (100, 101). The study further confirmed that inhibition of Nogo-A/NgR1 expression at the gene level or antagonism of its function at the protein level could reduce Rho A/ROCK signaling pathway activation and promote neurological recovery in post-stroke animals (97, 102). The same receptor complex and downstream mechanisms appear to be involved in the growth-inhibitory effects of other myelin-associated proteins, such as MAG and OMgp (103). NgR is mainly expressed in neuronal cytostomes and axons in the cerebral cortex, hippocampus, and dorsal root ganglia, as well as in activated microglia/macrophages in the CNS (101). There are three isoforms of NgR, namely NgR1, NgR2, and NgR3, among which NgR1 is the first receptor with high affinity to Nogo-66 in the extracellular segment of Nogo-A (104). The results of existing studies demonstrate that this receptor and its complex have a more direct effect on axon growth (105, 106). Neurogenesis was effectively promoted by inhibiting the expression of NgR1.TAT-NEP1–40, an antagonist of NgR1, can protect neurons and promote the recovery of neurological functions after stroke (107, 108).

Another mechanism by which Nogo-A exerts axonal growth inhibition is through binding to paired immunoglobulin-like receptor B (Pir B), which affects multiple protein functions involved in microfilament depolymerization and restriction of axon growth *via* downstream plenty of Src homology 3 domains (POSH) signaling molecules (109, 110). It has been shown that POSH formed an inhibitory complex by binding to F-actin-binding protein (Shroom3), which activated the POSH/Shroom3/ROCK signaling pathway, leading to a decrease in Myosin II expression and inhibition of axonal growth (111, 112). The study confirmed that the knockdown of Pir B caused more axon regeneration than the knockdown of NgR1, suggesting that Pir B plays a more important role in myelin inhibition (113). By antagonizing the action of Nogo-A and Pir B, it could inhibit POSH expression and suppress the activity of downstream molecules Shroom3/ROCK/Rho A, which effectively reverses the inhibition of its axonal growth (111, 114, 115). These results suggest that the Nogo-A/Pir B signaling pathway has an important role in axonal growth inhibition due to cerebral ischemia injury.

#### 3.1.2. MAG and OMgp

MAG is a member of the immunoglobulin superfamily. MAG is present in the preaxial membrane and unmyelinated regions of the CNS and PNS and is therefore well suited to interact with axonal receptors. It is both a ligand for axonal receptors required for the maintenance of myelinated axons and a receptor for axonal signals that promote oligodendrocyte differentiation, maintenance, and survival (116). Peripheral injection of a mouse monoclonal antibody against MAG resulted in significant preferential motor reinnervation in mice after transection of the femoral nerve, suggesting that interference with the rejection function of MAG facilitates the reinnervation of motor neurons to the correct pathway (117). It was also found that MAG levels could be reduced after MCAO, therefore mitigating axonal injury and improving neurological function in adult mice after cerebral ischemia (118).

OMgp is expressed not only through oligodendrocytes but also at high levels in various neurons. OMgp is the protein responsible for myelin partial inhibition, inducing growth cone collapse and inhibiting neurogenesis (119). Both MAG and OMgp interact with NGR with approximately the same relatively high affinity (120). There are relatively few studies on the effect of OMgp on axonal growth after stroke compared to Nogo-A and MAG.

### 3.2. Glial scar

Glial scar formation and altered astrocyte function are important pathological features of ischemic stroke. After a stroke, astrocytes proliferate reactively and later form a physical barrier of glial scarring with microglia, macrophages, and extracellular matrix (86). Glial fibrillary acidic protein (GFAP) is a characteristic marker of astrocyte activation and glial proliferation and constitutes a major component of the glial scar (121). The study has found that GFAP-positive reactive astrocytes significantly increased in the cortical infarct zone after ischemic stroke, resulting in enhanced expression of chondroitin sulfate proteoglycans (CSPGs) and formation of glial scar (86).

Glial scar is two-sided in nature. When an ischemic stroke occurs, the dense glial scar can isolate the area of injury from the surrounding tissues and impede the diffusion of large amounts of neurotoxic substances released from the infarcted area to the peripheral areas, thus effectively controlling further tissue infection, maintaining extracellular ion and fluid homeostasis, preventing overwhelming inflammatory and growth factor responses, and scavenging free radicals (122). However, in late cerebral ischemia, astrocytes increase in number, cytosolic hypertrophy, and protrusions increase and lengthen, creating a physical barrier in space that not only prevents reconnection between neurons but also works in concert with myelin-associated inhibitory factors to impede regeneration of injured axons, thus preventing recovery of CNS function in the chronic phase of ischemic stroke (86, 123). Astrocytes are able to upregulate several neuroinhibitory factors such as CSPGs, tyrosine-protein kinase-B2, and Slit protein C, impeding neuronal axon extension and synaptic regeneration, which is detrimental to the re-establishment of neural network structure and recovery of neurological function (123, 124).

It has been shown that CSPGs are the most important component of the glial scar that hinders regeneration after CNS injury, and their combination alone or with other extracellular matrix causes axonal extension toward the site of injury to stop at the glial scar, and that CSPGs reduce the plasticity of axonal growth (125). CSPG acts as an axon growth inhibitor by binding to PTPσ receptors to activate the downstream Rho A/ROCK pathway (12). Another study found that after nerve injury, the regeneration of injured axons and partial recovery of function was effectively promoted by eliminating CSPGs in the brain and spinal cord (125, 126). The above information suggests that reducing CSPGs has an important role in the recovery of motor function after a stroke.

## 4. Axon guidance cues

Species with bilateral symmetry possess a midline axis, a feature that becomes very important in vertebrates, especially humans (127). Newborn neurons have to decide whether or not to cross the midline or toward which direction they should send their axons to Flanagan and Van Vactor (128). Ramon y Cajal, the “father of neuroscience,” observed a very irregular structure at the distal end of the axon, which he called the “growth cone.” The growth cone is a very active structure composed mainly of cytoskeletal elements with high dynamics (e.g., microtubules, actin, and microfilaments) and numerous other proteins (129, 130). The growth cone is often equipped with one or more receptors to enable an appropriate response to axon guidance molecules that give developing neurons navigation to connect with distant targets (131, 132). In recent years, scientists have progressively confirmed the above theories and finally identified the classical guiding cues that give directionality to navigating axons: Netrins, Slits, Semaphorins (Sema), and Ephrins as well as their cognate receptors: deleted in colorectal cancer (DCC) and uncoordinated-5 (UNC5), Roundabout (Robo), Plexin and Neuropilin, and erythropoietin-producing hepatocellular (Eph), respectively (133, 134). Axon guidance molecules play a key role in the development of the nervous system and can regulate the regenerative capacity of neurons during neurological diseases.

The effects of Netrin on nerve axons are mainly manifested by inducing the migration of growth cones, orienting the axons, promoting the growth of growth cones, and prolonging the axons (135). Four types of Netrins were found in animal spinal cords: Netrin-1, Netrin-2, Netrin 3, and Netrin-4. Netrin-1 has both repulsive and attractive receptors. The UNC-5 homolog is a repulsive receptor that mediates the rejection of axons by Netrin-1 and inhibits the growth and extension of sensory nerve fibers; DCC is an attractive receptor that mediates the attraction of axons by Netrin-1 and promotes the growth and extension of sensory nerve fibers (135).

Slit directs the targeted growth of the growth cone through a concentration gradient (136). It is more sensitive to the action of peripheral protrusions of sensory neurons than to the action of central protrusions (137). Robo is the primary receptor for Slits and the Slit/Robo signaling pathway is one of the most important regulatory pathways for axon guidance, which is formed by the binding of Slit and Robo receptors (138). It mediates axonal rejection, neurogenesis, and migration during the development of the CNS and peripheral nervous system (138, 139).

Sema mediates axon guidance through chemical repulsion, and its receptors are mainly Neuropilins and plexins (140). Sema3A binds to its receptor Neuropilins-1, which not only reverses the direction of axonal growth, but also prevents the formation of axonal terminals and inhibits axonal extension (141).

The Eph receptor ligand is Ephrin. The Eph receptor and its ligand Ephrin are collectively known as the Eph family proteins (142). Ephrin- Eph signaling can regulate neuronal plasticity (143). Ephrin-A5 and EphA5 regulate the projection and location of nerve fibers (144). Ephrin-A5 was found to be induced in reactive astrocytes in the peri-infarct cortex and is an inhibitor of axonal sprouting and motor recovery in stroke patients (143).

**Figure 3:**
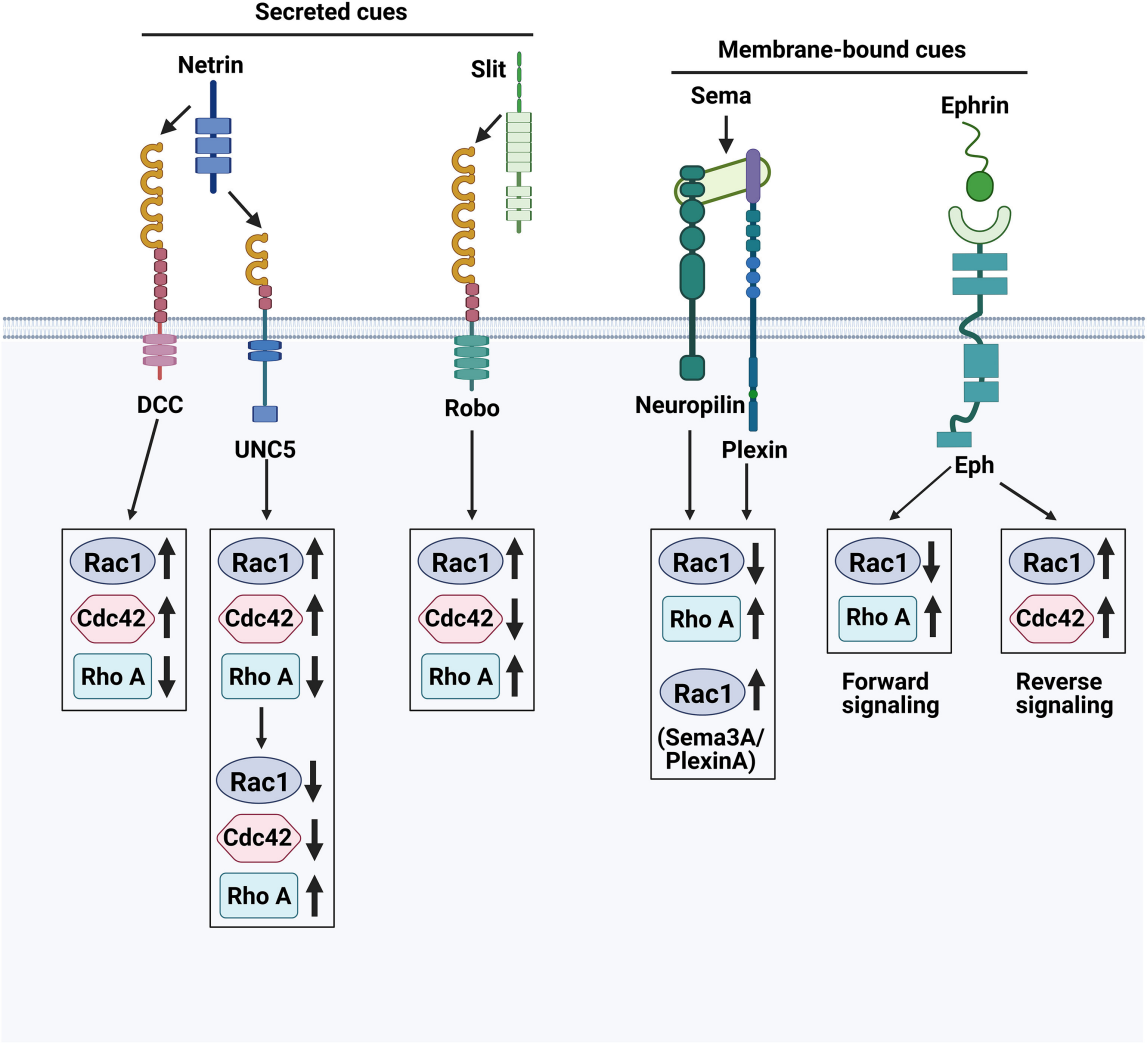
Axon guidance cues and their receptors. The growth cones at the axon tip are sensitive to repulsive and attractive guidance cues in their environment. The complex integration of these repulsive and attractive signals directs axons to their proper targets.

Rho GTPases are known for their role in the regulation of cell motility and cytoskeletal structure, with the most frequently studied members being mainly RhoA, Rac1 and Cdc42 (145). Studies have shown that activation of RhoA, Rac1, and Cdc42 can lead to the formation of different actin-based structures—respectively stress fibers, lamellipodia and filopodia (146). Different axon guidance cues can regulate axon regeneration by modulating these three molecules in response to changes in the microenvironment, as shown in Figure 3.

## 5. Acupuncture for post-stroke neurogenesis

Above summary of the factors affecting neurogenesis after stroke clearly demonstrates the complex response of the organism in the process of neurogenesis after injury. Targeting only one single factor for activation or inhibition thereby often results in an overkill situation and causes side effects (147, 148). From the perspective of treatment, there is an urgent need for stroke neuromodulation therapy without side effects, especially ones that can improve functional recovery by regulating the own regulatory mechanisms and facilitating brain repair of the body. Although as a traditional physical stimulation therapy, acupuncture is integral to modern medical practice and is considered a feasible and effective neural regulation strategy to improve functional recovery following stroke *via* the benign modulation of neuroplasticity (18, 19, 149). The research regarding the mechanism of effects of acupuncture on neurogenesis or axonal growth is accumulating (Table 1).

**Table 1:** Summary of the studies investigating acupuncture's effects on functional recovery improvement after stroke *via* regulating neurogenesis factors.

**Research group**	**Model**	**Acupoint**	**Acupuncture method**	**Stimulation parameter**	**Treatment course**	**Molecular and cellular results**	**Behavioral test**
Zhou (150)	MCAO	PC6, ST36	EA	2 Hz/30 Hz, 5 mA	30 min/day, 3, 7, 14, 28 days	BDNF↑, Sema3A↓, NRP-1↓	Bederson score
Kim et al. (151)	MCAO	GV20, GV14	EA	2 Hz, 2 V	20 min/day, 10 days from 5 days after MCAO	NSCs↑, BDNF↑, VEGF↑, p-PI3K↑, p-ERK↑	Morris Water Maze (MWM)
Kim et al. (152)	MCAO	GV20, GB7	EA	3 Hz for 5 s, with 2 s intervals, at an intensity of the muscle twitch threshold	5 min/2 days, 2 w from 3 days after MCAO	BDNF↑, TrkB↑	Garcia scale assessments
Yi et al. (153)	MCAO	GV20, GV14	EA	Disperse-dense wave, 5–10 times/s, intensity in the rat quiet tolerance degree, about 3–5 V	30 min/day, 2 or 5 weeks	P38↑, GAP-43↑, NGF↑, BDNF↑	/
Xia et al. (154)	I/R	KI3, LR3	MA	/	30 min/day, 14 days	BDNF↑, SYN↑, PSD↑	NSS, MWM
Zheng et al. (155)	I/R	GV20, GV24^+^	EA	2 Hz, 1 mA, 100 μs	10 min/day, 14 days	BDNF↑, TrkB↑, NMDAR1↑, AMPAR↑, GABA_A_R↑, CaMKII↑, NeuN↑, PSD-95↑	MWM, Novel Object Recognition Test, Open Field Test
Zhao et al. (156)	I/R	GV20, GV26	EA	2/100 Hz, 2 mA	40 min/day, 6 days	NGF↑	Zea Longa neurological score, MWM
Zhang et al. (157)	MCAO	nape cluster acupoints	MA	Twisting Angle is 60°	15 min/day, 15 days	BDNF↑, NGF↑	Zea Longa neurological score
Tao et al. (158)	I/R	LI11, ST36	EA	Dense disperse wave of 1 or 20-Hz	30 min/day, 3 days	BDNF↑, GFAP↑, vimentin↑, nestin↑, Cyclin D1↑, CDK4↑, phpspho-Rb↑	Zea Longa neurological score, Catwalk gait, Rotarod test
Gu et al. (159)	I/R	LI11, ST36	EA	2 Hz/15 Hz, 2–4 mA	20 min/day, 7 days	BDNF↑, PGC-1α↑, FNDC5↑	Zea Longa neurological score, Balance Beam score
Chen et al. (160)	I/R	LI11, ST36	EA	Disperse wave of 1 and 20 Hz	30 min, once	BDNF↑, GDNF↑, PI3K↑, p-Akt↑, t-Akt↑, Bcl-2/Bax ratio↑	Zea Longa neurological score
Xue et al. (161)	I/R	LI11, ST36	EA	Disperse wave of 4 and 20 Hz	30 min/day, 3 days	PI3K↑, p-Akt↑, p-Bad↑, Bcl-2↑, Bax↓, cleaved caspase−3↓	Zea Longa neurological score
Yang et al. (162)	MCAO	GV16, GV8	EA	60 Hz 1 s and 2 Hz 3 s alternately at an intensity of 10 mA	20 min, once	BrdU+ cells↑, BrdU+/CRMP-4(+)↑, BrdU+/MAP-2(+)↑	/
Tao et al. (163)	MCAO	LI11, ST36	EA	1 Hz/20 Hz, at an intensity of the muscle twitch threshold (the muscle twitch threshold was about 0.01 mA)	30 min/day, 7 days	BDNF↑, Nestin↑, Notch1↑, NICD↑, Hes1↑, GDNF↑, D1↑, Cdk4↑, p-Rb↑, p21↓, p27↓	Zea Longa neurological score
Chen et al. (164)	MCAO	LI11, ST36	EA	disperse-dense waves of 1 or 20 Hz frequencies	30 min/day, 3 days	GFAP↑, Nestin↑, Wnt1↑, β-catenin↑, GSK3↓	Zea Longa neurological score
Zhang et al. (165)	MCAO	LI11, ST36	EA	1/20 Hz, 1 mA	30 min/day, 21 days	miR-146b↑, NeuroD1↑	mNSS
Zhao et al. (166)	MCAO	GV20	EA	1–2 mA, dense-disperse frequency of 2/10 Hz	30 min/day, 5 days	miR-132↑, SOX2↓	The rotarod test, limb placement test, body swing test, measurement of forelimb placing
Xu et al. (167)	MCAO	GV26, GV20	EA	3.85 Hz/6.25 Hz, 0.8–1.3 mA	30 min/day, 3 days	miR-210↑, CD34↑, HIF-1α↑, VEGF↑	Zea Longa neurological score
Tan et al. (168)	I/R	GV20, GV14	EA	3 V, 3 Hz	15 min/day, 28 days	NgR↓	Zea Longa neurological score
Xiao et al. (169)	MCAO	PC2, PC3, PC6, PC7	EA	2–4 V	30 min/day, 21 days	Nogo-A↓, NgR↓	Zea Longa neurological score
Chen et al. (170)	I/R	GV20, GV14	EA	Continuous-wave stimulation at 2 Hz (intensity, 1 mA)	30 min/day, 7 days	GAP43↑, BDNF↑, RhoA↓, ROCK↓	/
Huang et al. (171)	MCAO	LI11, ST36	EA	A dense disperse wave of 1 and 20 Hz (adjusted to the muscle twitch threshold)	30 min/day, 3, 7, 14 days	GAP43↑, RhoA↓, ROCK↓, Nogo-A↓, NgR↓	Zea Longa neurological score

Neiguan (PC6), Zusanli (ST36), Baihui (GV20), Dazhui (GV14), Taixi (KI3), Taichong (LR3), Yintang (GV24^+^), Shuigou (GV26), Quchi (LI11), Jinsuo (GV8), Fengfu (GV16), Tianquan (PC2), Quze (PC3), Daling (PC7).

### 5.1. Acupuncture for enhancing factors

**Figure 4:**
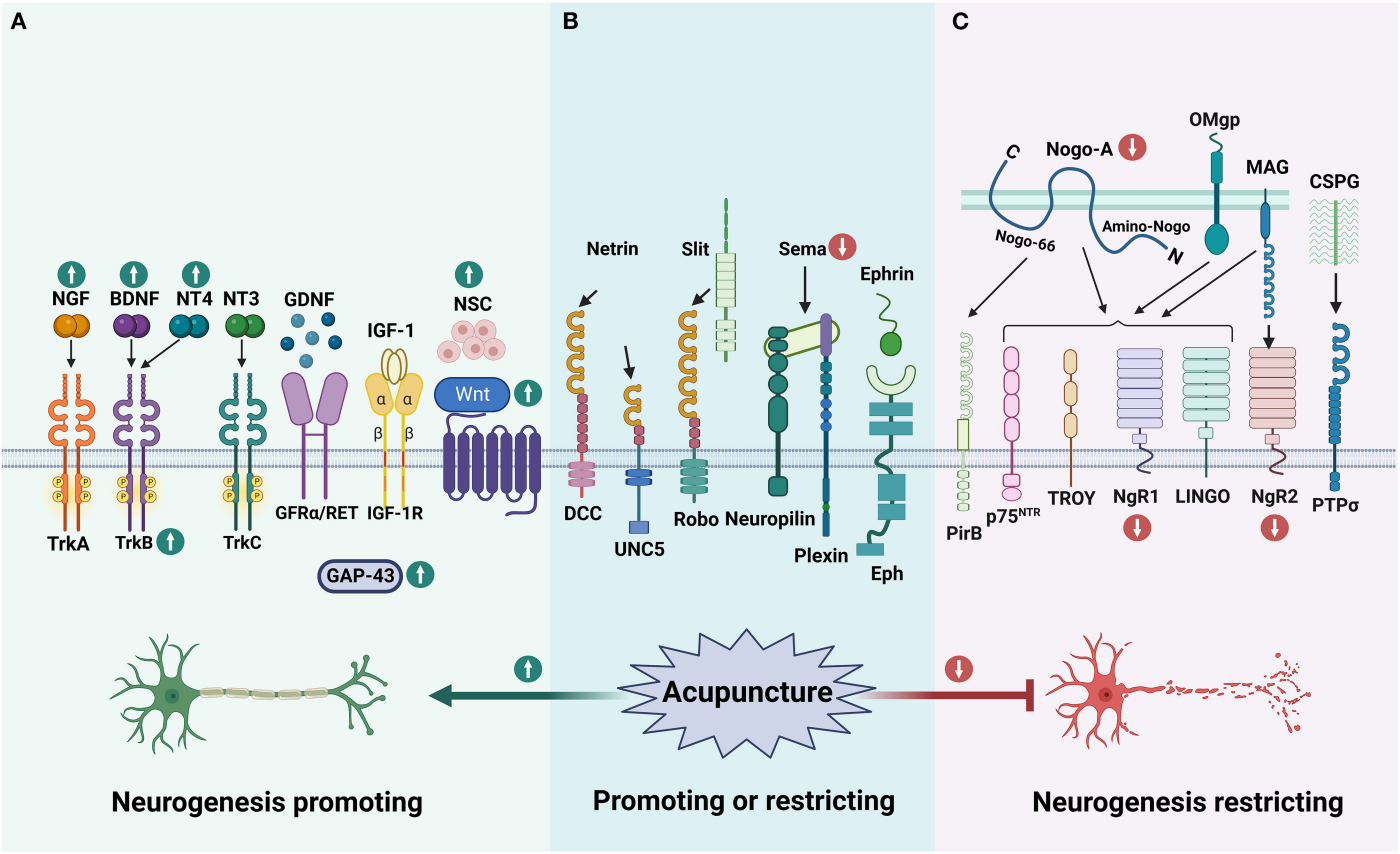
The effect of acupuncture on neurogenesis impact factors. **(A)** Shows enhancers for neurogenesis, **(B)** illustrates guidance cues, and **(C)** shows inhibitors. According to the currently limited studies, acupuncture promotes neurogenesis and functional recovery after stroke by upregulating some of the enhancing factors of neurogenesis and downregulating the inhibiting factors that affect neurogenesis. The green up arrow represents up-regulation, and the red down arrow represents down-regulation.

Electroacupuncture (EA) can improve the symptoms of neurological deficits and promote the recovery of motor function in post-stroke rats. The mechanism may be related to the upregulation of BDNF by EA at *Neiguan* (PC6) and *Zusanli* (ST36) to promote neuronal growth, and the downregulation of Sema3A and NRP-1 to reduce the inhibitory effect on axonal regeneration (150) (Figures 4A, B). Studies have shown that EA at *Baihui* (GV20) and *Dazhui* (GV14) significantly improved functional recovery by enhancement of proliferation and differentiation of NSCs *via* upregulating the BDNF and TrkB expression (151, 152). In addition, EA may promote synaptic plasticity after stroke by protecting and improving synaptic ultrastructure in the rat ischemic cerebral cortex and increasing the expression of synaptophysin P38, GAP-43, NGF and BDNF (153). Manual acupuncture (MA) at *Taixi* (KI3) and *Taichong* (LR3) can promote functional recovery as well as learning and memory abilities after ischemic stroke by enhancing BDNF and SYN expression and synaptic structural reconstruction in the ipsilateral hippocampus after I/R (154). EA on trigeminal innervation points [GV20 and *Yintang* (GV24^+^)] is an effective therapy for poststroke cognitive impairment and is associated with neuroprotection and synaptic plasticity-mediated in relevant brain regions in the MCAO rat model (155). EA reversed I/R injury-induced BDNF, TrkB, N-methyl-D-aspartate receptor 1 (NMDAR1), α-amino-3-hydroxy-5-methyl-4-isoxazole propionic acid receptor (AMPAR), γ-aminobutyric acid type A receptor, Ca^2+^/calmodulin-dependent protein kinase II (CaMKII), neuronal nucleus (NeuN) and postsynaptic density protein 95 (PSD-95) expressed in the prefrontal cortex and hippocampus.

It was also found that EA at GV20 and *Shuigou* (GV26) enhanced the permeability of the blood-brain barrier in the prefrontal cortex and induced the uptake of NGF by prefrontal neurons (156). Nape cluster acupuncture has neuroprotective and restorative effects in rats with post-ischemic stroke sequelae, and its mechanism may involve effective upregulation of BDNF and NGF protein expression (157). EA treatment at *Quchi* (LI11) and ST36 acupoints exerted neuroprotective effects on I/R-injured rats through the proliferation of GFAP/vimentin/nestin-positive reactive astrocytes and secretion of potentially reactive astrocyte-derived BDNF and may also be related to the activation of cortical PGC-1α/Irisin (FNDC5)/BDNF pathway by electroacupuncture (158, 159). In addition, EA ST36 and LI11 could also increase the expression of PI3K, p-Akt, p-Bad, Bcl-2, BDNF, and GDNF, which exerted neuroprotective effects by activating the PI3K/Akt pathway (160, 161) (Figure 4A).

EA at *Jinsuo* (GV8) and *Fengfu* (GV16) caused proliferating endogenous NSCs to migrate from the SVZ region to the damaged area of the striatum in rats' post-stroke, thereby promoting neurogenesis in the brain striatum (162). EA promotes the proliferation and differentiation of endogenous NSCs by upregulating the Wnt/β-linked protein signaling pathway and the secretion of neurotrophic factors, thereby ameliorating neurological defects and producing a therapeutic effect on cerebral ischemia (163, 164) (Figure 4A).

EA at LI11 and ST36 can promote endogenous NSCs differentiation *via* exosome-mediated miR-146b, thereby improving neurological dysfunction after ischemic stroke (165). EA at GV20 enhances neurobehavioral functional recovery against ischemic stroke *via* targeting of SOX2-mediated axonal regeneration by miR-132 (166). EA at GV26 and GV20 could activate the HIF-1α/VEGF/Notch 1 signal pathway to facilitate angiogenesis after ischemic stroke *via* exosomal miR-210 (167).

### 5.2. Acupuncture for inhibiting factors

The protective effect of EA at GV14 and GV20 on hypertensive I/R cerebral injury rats might be closely related to down-regulating central nervous system myelin growth inhibit ion mediated factors Nogo-A receptor NgR protein expression (168). EA stimulation of acupoints of pericardium meridian can down-regulate the expressions of cerebral Nogo-A and NgR1 mRNA in cerebral ischemia rats, which is conducive to nerve repair after cerebral ischemia (169). EA can downregulate the RhoA/ROCK pathway to promote axonal regeneration (170). EA at LI11 and ST36 could significantly improve neurological deficit scores following stroke *via* inhibited Nogo-A/NgR/RhoA/ROCK signaling (171) (Figure 4C).

Taken together, acupuncture can promote neurogenesis after stroke by enhancing axonal growth factors as well as decreasing axonal growth inhibitory factors, thus promoting neurological recovery. More and more in-depth studies are worthwhile to enrich the multi-target mechanism of clinical acupuncture for post-stroke motor dysfunction.

## 6. Summary and prospect

The difficulty of central neurogenesis after cerebral ischemia in adults is the main cause of neurological dysfunction after stroke and promoting neurogenesis after stroke has become a hot button in cerebrovascular disease research in recent years. Promoting axonal sprouting, synaptic remodeling, and suppressing the central damage microenvironment will be an important pathway to improve neurological impairment after ischemia, which is also a key issue in stroke treatment.

Acupuncture can regulate post-ischemic neurogenesis at multiple levels and targets and can promote the expression of post-ischemic NSCs and other factors that are beneficial to neurogenesis. On the other hand, acupuncture can reduce the local inhibitory microenvironment in the injured center, providing a favorable microenvironment for neurogenesis and repair. However, the specific mechanisms of these factors are not yet fully understood, because the mechanism of neurogenesis after cerebral ischemia is complex and influenced by various factors, and most of the studies on the mechanism of neurogenesis after stroke are focused on the enhancing factors of neurogenesis (such as growth factors), fewer studies on the effects of acupuncture on neurotransmitters, related inhibitory proteins and axon guidance cues. In the future, studies investigating the role of acupuncture on neurogenesis after stroke can particularly focus on axonal inhibitory factors and the complete pathway, which can better illustrate the mechanism of acupuncture initiation.

It appears that more researches have been done on EA than on MA, probably related to the stronger stimulation of EA and the easier control and fixation of stimulation parameters (172). Generally, an important factor of the effectiveness of acupuncture in clinical practice is the needling technique and MA is more commonly used in the clinic. However, there is a lack of research on the effects of MA on neuroplasticity. Future studies could attempt to compare the effects of MA and EA on neurogenesis to provide greater clinical guidance for acupuncture.

The acupoints used in each study varied. The most frequently applied acupoints are GV20, GV14, and ST36 (Table 1), which are located on Governor Vessel (GV) and stomach meridian. The GV runs along the middle of the back and connects to the brain, so points on GV are commonly used for brain disorders. The stomach meridian is connected to the stomach organ and is often considered to be the origin of energy, so points on this meridian are often used for conditions in which the body is deprived of energy. Although these three points are frequently used, few studies have compared them with each other or with other acupoints to determine which acupoint or combination of points produces the best effect on neuroplasticity after cerebral ischemia. In addition, differences in the frequency, intensity, and treatment course of EA might produce a variety of effects on neuroplasticity. Although many studies have used dense/sparse wave stimulation (Table 1), the number of studies comparing different stimulation parameters is relatively small. Therefore, both optimal stimulation conditions and therapeutic time windows for acupuncture need to be based on additional and more solid mechanistic studies in order to be supported by reliable data from preclinical studies.

Finally, most of the current acupuncture interventions have been studied through animal experiments, and there is a lack of large sample clinical studies, and further research is needed on how to effectively apply them to clinical practice. Therefore, in the future, we should carry out research on the interaction between various factors by acupuncture treatment. Using neuroimaging and other technical means, combine animal experiments with clinical practice to provide a theoretical basis for clinical acupuncture treatment of stroke.

## 7. Conclusion

The global burden of stroke is increasing every year due to population growth and aging trends. Stroke has become the most significant risk factor for human health worldwide. We reviewed studies on the mechanisms of neurogenesis after stroke, analyzed the role of various common factors on neurogenesis, and discussed the effects of acupuncture on neurogenesis and functional recovery after stroke. Stroke-induced neuroplasticity is a promising therapeutic target because it allows the brain in injured areas to re-establish neural connections and heal the damage caused by ischemia. Due to the complex interactions between various factors affecting neurogenesis, interfering with one factor alone often leads to an overkill situation. Therefore, there is still a global need to develop better treatment options without side effects. Acupuncture is an ancient physical stimulation therapy that has been practiced in China for thousands of years and provides a benign regulation of the body through the stimulation of body acupoints. Acupuncture can promote functional recovery after stroke, and its mechanism of action is based on the modulation of neuroplasticity. However, there is still a lack of more comprehensive mechanistic evidence to fully demonstrate the role of acupuncture in the neurogenesis microenvironment.

In the future, based on neurogenesis mechanisms, the experimental design can focus on screening optimal factors of acupuncture treatment, particularly appropriate intervention time, needling techniques, acupoints, and acupuncture sessions, so as to provide more reliable mechanistic evidence for acupuncture strategy in functional rehabilitation after stroke.

## Author contributions

Manuscript writing: J-DM and L-XM. Conception and design: J-DM, L-XM, ZZ, XQ, Q-YZ, L-HM, and T-YS. All authors conceived and approved the final manuscript.

## References

[1] GBD 2019 Stroke Collaborators. Global, regional, and national burden of stroke and its risk factors, 1990-2019: a systematic analysis for the Global Burden of Disease Study 2019. Lancet Neurol. (2021) 20:795–820. 10.1016/S1474-4422(21)00252-0 34487721 PMC8443449

[2] Owolabi MO Thrift AG Mahal A Ishida M Martins S Johnson WD. Primary stroke prevention worldwide: translating evidence into action. Lancet Public Health. (2022) 7:e74–85. 10.1016/S2468-2667(21)00230-9 34756176 PMC8727355

[3] Veerbeek JM Kwakkel G van Wegen EE Ket JC Heymans MW. Early prediction of outcome of activities of daily living after stroke: a systematic review. Stroke. (2011) 42:1482–8. 10.1161/STROKEAHA.110.604090 21474812

[4] Kantak SS Stinear JW Buch ER Cohen LG. Rewiring the brain: potential role of the premotor cortex in motor control, learning, and recovery of function following brain injury. Neurorehabil Neural Repair. (2012) 26:282–92. 10.1177/1545968311420845 21926382 PMC4886541

[5] Ramic M Emerick AJ Bollnow MR O'Brien TE Tsai SY Kartje GL. Axonal plasticity is associated with motor recovery following amphetamine treatment combined with rehabilitation after brain injury in the adult rat. Brain Res. (2006) 1111:176–86. 10.1016/j.brainres.2006.06.063 16920088

[6] Hatakeyama M Ninomiya I Kanazawa M. Angiogenesis and neuronal remodeling after ischemic stroke. Neural Regen Res. (2020) 15:16–9. 10.4103/1673-5374.264442 31535636 PMC6862417

[7] Lo EH. Degeneration and repair in central nervous system disease. Nat Med. (2010) 16:1205–9. 10.1038/nm.2226 21052074 PMC3985732

[8] Joy MT Carmichael ST. Encouraging an excitable brain state: mechanisms of brain repair in stroke. Nat Rev Neurosci. (2021) 22:38–53. 10.1038/s41583-020-00396-7 33184469 PMC10625167

[9] Liu J Yang X Jiang L Wang C Yang M. Neural plasticity after spinal cord injury. Neural Regen Res. (2012) 7:386–91. 10.3969/j.issn.1673-5374.2012.05.010 25774179 PMC4350123

[10] Hickmott PW Ethell IM. Dendritic plasticity in the adult neocortex. Neuroscientist. (2006) 12:16–28. 10.1177/1073858405282417 16394190

[11] Balu DT Lucki I. Adult hippocampal neurogenesis: regulation, functional implications, and contribution to disease pathology. Neurosci Biobehav Rev. (2009) 33:232–52. 10.1016/j.neubiorev.2008.08.007 18786562 PMC2671071

[12] Luo F Wang J Zhang Z You Z Bedolla A Okwubido-Williams F. Inhibition of CSPG receptor PTPσ promotes migration of newly born neuroblasts, axonal sprouting, and recovery from stroke. Cell Rep. (2022) 40:111137. 10.1016/j.celrep.2022.111137 35905716 PMC9677607

[13] Globus A Rosenzweig MR Bennett EL Diamond MC. Effects of differential experience on dendritic spine counts in rat cerebral cortex. J Comp Physiol Psychol. (1973) 82:175–81. 10.1037/h0033910 4571892

[14] Kempermann G Kuhn HG Gage FH. More hippocampal neurons in adult mice living in an enriched environment. Nature. (1997) 386:493–5. 10.1038/386493a0 9087407

[15] Hesketh T Zhu WX. Health in China. Traditional Chinese medicine: one country, two systems. BMJ. (1997) 315:115–7. 10.1136/bmj.315.7100.115 9240055 PMC2127090

[16] Andersson S Lundeberg T. Acupuncture–from empiricism to science: functional background to acupuncture effects in pain and disease. Med Hypotheses. (1995) 45:271–81. 10.1016/0306-9877(95)90117-5 8569551

[17] Kagitani F Uchida S Hotta H. Afferent nerve fibers and acupuncture. Auton Neurosci. (2010) 157:2–8. 10.1016/j.autneu.2010.03.004 20494626

[18] Xiong J Zhang Z Ma Y Li Z Zhou F Qiao N. The effect of combined scalp acupuncture and cognitive training in patients with stroke on cognitive and motor functions. NeuroRehabilitation. (2020) 46:75–82. 10.3233/NRE-192942 32039871

[19] Hu X Li B Wang X. Scalp acupuncture therapy combined with exercise can improve the ability of stroke patients to participate in daily activities. Complement Ther Clin Pract. (2021) 43:101343. 10.1016/j.ctcp.2021.101343 33714169

[20] Bond AM Ming GL Song H. Adult mammalian neural stem cells and neurogenesis: five decades later. Cell Stem Cell. (2015) 17:385–95. 10.1016/j.stem.2015.09.003 26431181 PMC4683085

[21] Zhao C Deng W Gage FH. Mechanisms and functional implications of adult neurogenesis. Cell. (2008) 132:645–60. 10.1016/j.cell.2008.01.033 18295581

[22] Gage FH. Mammalian neural stem cells. Science. (2000) 287:1433–8. 10.1126/science.287.5457.1433 10688783

[23] Tang Y Wang J Lin X Wang L Shao B Jin K. Neural stem cell protects aged rat brain from ischemia-reperfusion injury through neurogenesis and angiogenesis. J Cereb Blood Flow Metab. (2014) 34:1138–47. 10.1038/jcbfm.2014.61 24714034 PMC4083376

[24] Zhang ZG Chopp M. Neurorestorative therapies for stroke: underlying mechanisms and translation to the clinic. Lancet Neurol. (2009) 8:491–500. 10.1016/S1474-4422(09)70061-4 19375666 PMC2727708

[25] Liu X Ye R Yan T Yu SP Wei L Xu G. Cell based therapies for ischemic stroke: from basic science to bedside. Prog Neurobiol. (2014) 115:92–115. 10.1016/j.pneurobio.2013.11.007 24333397 PMC4038267

[26] Nih LR Deroide N Leré-Déan C Lerouet D Soustrat M Levy BI. Neuroblast survival depends on mature vascular network formation after mouse stroke: role of endothelial and smooth muscle progenitor cell co-administration. Eur J Neurosci. (2012) 35:1208–17. 10.1111/j.1460-9568.2012.08041.x 22512253

[27] Mangin G Kubis N. Cell therapy for ischemic stroke: how to turn a promising preclinical research into a successful clinical story. Stem Cell Rev Rep. (2019) 15:176–93. 10.1007/s12015-018-9864-3 30443706

[28] Shen H Gu X Wei ZZ Wu A Liu X Wei L. Combinatorial intranasal delivery of bone marrow mesenchymal stem cells and insulin-like growth factor-1 improves neurovascularization and functional outcomes following focal cerebral ischemia in mice. Exp Neurol. (2021) 337:113542. 10.1016/j.expneurol.2020.113542 33275952

[29] Kowiański P Lietzau G Czuba E Waśkow M Steliga A Moryś J. A key factor with multipotent impact on brain signaling and synaptic plasticity. Cell Mol Neurobiol. (2018) 38:579–93. 10.1007/s10571-017-0510-4 28623429 PMC5835061

[30] Santos D González-Pérez F Giudetti G Micera S Udina E Del Valle J. Preferential enhancement of sensory and motor axon regeneration by combining extracellular matrix components with neurotrophic factors. Int J Mol Sci. (2016) 18:65. 10.3390/ijms18010065 28036084 PMC5297700

[31] O'Donovan KJ. Intrinsic axonal growth and the drive for regeneration. Front Neurosci. (2016) 10:486. 10.3389/fnins.2016.00486 27833527 PMC5081384

[32] Lake D Corrêa SA Müller J. Negative feedback regulation of the ERK1/2 MAPK pathway. Cell Mol Life Sci. (2016) 73:4397–413. 10.1007/s00018-016-2297-8 27342992 PMC5075022

[33] Zhang Y Yang M Yuan Q He Q Ping H Yang J. Piperine ameliorates ischemic stroke-induced brain injury in rats by regulating the PI3K/AKT/mTOR pathway. J Ethnopharmacol. (2022) 295:115309. 10.1016/j.jep.2022.115309 35597410

[34] Wang M Hayashi H Horinokita I Asada M Iwatani Y Liu JX. Neuroprotective effects of Senkyunolide I against glutamate-induced cells death by attenuating JNK/caspase-3 activation and apoptosis. Biomed Pharmacother. (2021) 140:111696. 10.1016/j.biopha.2021.111696 34044281

[35] Atwal JK Massie B Miller FD Kaplan DR. The TrkB-Shc site signals neuronal survival and local axon growth *via* MEK and P13-kinase. Neuron. (2000) 27:265–77. 10.1016/S0896-6273(00)00035-0 10985347

[36] Kang DS Yang YR Lee C Kim S Ryu SH Suh PG. Roles of phosphoinositide-specific phospholipase Cγ1 in brain development. Adv Biol Regul. (2016) 60:167–73. 10.1016/j.jbior.2015.10.002 26588873

[37] Sofroniew MV Howe CL Mobley WC. Nerve growth factor signaling, neuroprotection, and neural repair. Annu Rev Neurosci. (2001) 24:1217–81. 10.1146/annurev.neuro.24.1.1217 11520933

[38] Marlin MC Li G. Biogenesis and function of the NGF/TrkA signaling endosome. Int Rev Cell Mol Biol. (2015) 314:239–57. 10.1016/bs.ircmb.2014.10.002 25619719 PMC4307610

[39] Sainath R Gallo G. Bioenergetic requirements and spatiotemporal profile of nerve growth factor induced pi3k-akt signaling along sensory axons. Front Mol Neurosci. (2021) 14:726331. 10.3389/fnmol.2021.726331 34630035 PMC8497901

[40] Biarc J Chalkley RJ Burlingame AL Bradshaw RA. Dissecting the roles of tyrosines 490 and 785 of TrkA protein in the induction of downstream protein phosphorylation using chimeric receptors. J Biol Chem. (2013) 288:16606–18. 10.1074/jbc.M113.475285 23589303 PMC3675596

[41] Bradshaw RA Chalkley RJ Biarc J Burlingame AL. Receptor tyrosine kinase signaling mechanisms: devolving TrkA responses with phosphoproteomics. Adv Biol Regul. (2013) 53:87–96. 10.1016/j.jbior.2012.10.006 23266087 PMC3577974

[42] Sugimoto T Kuroda H Horii Y Moritake H Tanaka T Hattori S. Signal transduction pathways through TRK-A and TRK-B receptors in human neuroblastoma cells. Jpn J Cancer Res. (2001) 92:152–60. 10.1111/j.1349-7006.2001.tb01077.x 11223544 PMC5926689

[43] Cai J Hua F Yuan L Tang W Lu J Yu S. Potential therapeutic effects of neurotrophins for acute and chronic neurological diseases. Biomed Res Int. (2014) 2014:601084. 10.1155/2014/601084 24818146 PMC4000962

[44] Zhao H Zhang Y Zhang Y Shen Y Zhang Y Bi F. NGF/FAK signal pathway is implicated in angiogenesis after acute cerebral ischemia in rats. Neurosci Lett. (2018) 672:96–102. 10.1016/j.neulet.2018.02.023 29458087

[45] Ahmed S Kwatra M Gawali B Panda SR Naidu V. Potential role of TrkB agonist in neuronal survival by promoting CREB/BDNF and PI3K/Akt signaling *in vitro* and *in vivo* model of 3-nitropropionic acid (3-NP)-induced neuronal death. Apoptosis. (2021) 26:52–70. 10.1007/s10495-020-01645-x 33226552

[46] Lin G Zhang H Sun F Lu Z Reed-Maldonado A Lee YC. Brain-derived neurotrophic factor promotes nerve regeneration by activating the JAK/STAT pathway in Schwann cells. Transl Androl Urol. (2016) 5:167–75. 10.21037/tau.2016.02.03 27141442 PMC4837308

[47] Gaidin SG Turovskaya MV Gavrish MS Babaev AA Mal'tseva VN Blinova EV. The selective BDNF overexpression in neurons protects neuroglial networks against OGD and glutamate-induced excitotoxicity. Int J Neurosci. (2020) 130:363–83. 10.1080/00207454.2019.1691205 31694441

[48] Li J Zhang Q Li S Niu L Ma J Wen L. α7nAchR mediates transcutaneous auricular vagus nerve stimulation-induced neuroprotection in a rat model of ischemic stroke by enhancing axonal plasticity. Neurosci Lett. (2020) 730:135031. 10.1016/j.neulet.2020.135031 32416113

[49] Jean I Lavialle C Barthelaix-Pouplard A Fressinaud C. Neurotrophin-3 specifically increases mature oligodendrocyte population and enhances remyelination after chemical demyelination of adult rat CNS. Brain Res. (2003) 972:110–8. 10.1016/S0006-8993(03)02510-1 12711083

[50] Omar NA Kumar J Teoh SL. Neurotrophin-3 and neurotrophin-4: the unsung heroes that lies behind the meninges. Neuropeptides. (2022) 92:102226. 10.1016/j.npep.2022.102226 35030377

[51] Bothwell M. NGF, BDNF, NT3, and NT4. Handb Exp Pharmacol. (2014) 220:3–15. 10.1007/978-3-642-45106-5_1 24668467

[52] Harvey AR Lovett SJ Majda BT Yoon JH Wheeler LP Hodgetts SI. Neurotrophic factors for spinal cord repair: which, where, how and when to apply, and for what period of time. Brain Res. (2015) 1619:36–71. 10.1016/j.brainres.2014.10.049 25451132

[53] Duricki DA Drndarski S Bernanos M Wood T Bosch K Chen Q. Stroke recovery in rats after 24-hour-delayed intramuscular neurotrophin-3 infusion. Ann Neurol. (2019) 85:32–46. 10.1002/ana.25386 30525223 PMC6492080

[54] Poduslo JF Curran GL. Permeability at the blood-brain and blood-nerve barriers of the neurotrophic factors: NGF, CNTF, NT-3, BDNF. Brain Res Mol Brain Res. (1996) 36:280–6. 10.1016/0169-328X(95)00250-V 8965648

[55] Logan A Ahmed Z Baird A Gonzalez AM Berry M. Neurotrophic factor synergy is required for neuronal survival and disinhibited axon regeneration after CNS injury. Brain. (2006) 129:490–502. 10.1093/brain/awh706 16339795

[56] Endres M Fan G Hirt L Fujii M Matsushita K Liu X. Ischemic brain damage in mice after selectively modifying BDNF or NT4 gene expression. J Cereb Blood Flow Metab. (2000) 20:139–44. 10.1097/00004647-200001000-00018 10616802

[57] Chung JY Kim MW Bang MS Kim M. Increased expression of neurotrophin 4 following focal cerebral ischemia in adult rat brain with treadmill exercise. PLoS ONE. (2013) 8:e52461. 10.1371/journal.pone.0052461 23526925 PMC3601124

[58] Bespalov MM Saarma M GDNF. family receptor complexes are emerging drug targets. Trends Pharmacol Sci. (2007) 28:68–74. 10.1016/j.tips.2006.12.005 17218019

[59] Sariola H Saarma M. Novel functions and signalling pathways for GDNF. J Cell Sci. (2003) 116:3855–62. 10.1242/jcs.00786 12953054

[60] Lundgren TK Nakahata K Fritz N Rebellato P Zhang S Uhlén P. PLCγ phosphotyrosine binding domain regulates Ca^2+^ signaling and neocortical neuronal migration. PLoS ONE. (2012) 7:e31258. 10.1371/journal.pone.0031258 22355350 PMC3280273

[61] Liu Y Wang S Luo S Li Z Liang F Zhu Y. Intravenous PEP-1-GDNF is protective after focal cerebral ischemia in rats. Neurosci Lett. (2016) 617:150–5. 10.1016/j.neulet.2016.02.017 26876444

[62] Beker M Caglayan AB Beker MC Altunay S Karacay R Dalay A. Lentivirally administered glial cell line-derived neurotrophic factor promotes post-ischemic neurological recovery, brain remodeling and contralesional pyramidal tract plasticity by regulating axonal growth inhibitors and guidance proteins. Exp Neurol. (2020) 331:113364. 10.1016/j.expneurol.2020.113364 32454038

[63] Duarte Azevedo M Sander S Tenenbaum L. GDNF, a neuron-derived factor upregulated in glial cells during disease. J Clin Med. (2020) 9:456. 10.3390/jcm9020456 32046031 PMC7073520

[64] Grasselli G Strata P. Structural plasticity of climbing fibers and the growth-associated protein GAP-43. Front Neural Circuits. (2013) 7:25. 10.3389/fncir.2013.00025 23441024 PMC3578352

[65] Benowitz LI Routtenberg A. GAP-43: an intrinsic determinant of neuronal development and plasticity. Trends Neurosci. (1997) 20:84–91. 10.1016/S0166-2236(96)10072-2 9023877

[66] Aigner L Caroni P. Absence of persistent spreading, branching, and adhesion in GAP-43-depleted growth cones. J Cell Biol. (1995) 128:647–60. 10.1083/jcb.128.4.647 7860637 PMC2199882

[67] Carmichael ST Archibeque I Luke L Nolan T Momiy J Li S. Growth-associated gene expression after stroke: evidence for a growth-promoting region in peri-infarct cortex. Exp Neurol. (2005) 193:291–311. 10.1016/j.expneurol.2005.01.004 15869933

[68] Han MH Jiao S Jia JM Chen Y Chen CY Gucek M. The novel caspase-3 substrate Gap43 is involved in AMPA receptor endocytosis and long-term depression. Mol Cell Proteomics. (2013) 12:3719–31. 10.1074/mcp.M113.030676 24023391 PMC3861719

[69] Wang JY Chen F Fu XQ Ding CS Zhou L Zhang XH. Caspase-3 cleavage of dishevelled induces elimination of postsynaptic structures. Dev Cell. (2014) 28:670–84. 10.1016/j.devcel.2014.02.009 24631402

[70] Gorup D Bohaček I Miličević T Pochet R Mitrečić D KriŽ J. Increased expression and colocalization of GAP43 and CASP3 after brain ischemic lesion in mouse. Neurosci Lett. (2015) 597:176–82. 10.1016/j.neulet.2015.04.042 25929184

[71] Huat TJ Khan AA Pati S Mustafa Z Abdullah JM Jaafar H. IGF-1 enhances cell proliferation and survival during early differentiation of mesenchymal stem cells to neural progenitor-like cells. BMC Neurosci. (2014) 15:91. 10.1186/1471-2202-15-91 25047045 PMC4117972

[72] Castilla-Cortázar I Iturrieta I García-Magariño M Puche JE Martín-Estal I Aguirre GA. Neurotrophic factors and their receptors are altered by the mere partial IGF-1 deficiency. Neuroscience. (2019) 404:445–58. 10.1016/j.neuroscience.2019.01.041 30708048

[73] Prager D Melmed S. Insulin and insulin-like growth factor I receptors: are there functional distinctions. Endocrinology. (1993) 132:1419–20. 10.1210/endo.132.4.8462444 8462444

[74] Rechler MM Nissley SP. The nature and regulation of the receptors for insulin-like growth factors. Annu Rev Physiol. (1985) 47:425–42. 10.1146/annurev.ph.47.030185.002233 2986537

[75] Xu GJ Cai S Wu JB. Effect of insulin-like growth factor-1 on bone morphogenetic protein-2 expression in hepatic carcinoma SMMC7721 cells through the p38 MAPK signaling pathway. Asian Pac J Cancer Prev. (2012) 13:1183–6. 10.7314/APJCP.2012.13.4.1183 22799302

[76] Escott GM Jacobus AP Loss ES. PI3K-dependent actions of insulin and IGF-I on seminiferous tubules from immature rats. Pflugers Arch. (2013) 465:1497–505. 10.1007/s00424-013-1287-z 23636775

[77] Zhu W Fan Y Frenzel T Gasmi M Bartus RT Young WL. Insulin growth factor-1 gene transfer enhances neurovascular remodeling and improves long-term stroke outcome in mice. Stroke. (2008) 39:1254–61. 10.1161/STROKEAHA.107.500801 18309153 PMC2553752

[78] Forró T Bajkó Z Băla?a A Băla?a R. Dysfunction of the neurovascular unit in ischemic stroke: highlights on micrornas and exosomes as potential biomarkers and therapy. Int J Mol Sci. (2021) 22:5621. 10.3390/ijms22115621 34070696 PMC8198979

[79] Lu XC Zheng JY Tang LJ Huang BS Li K Tao Y. MiR-133b Promotes neurite outgrowth by targeting RhoA expression. Cell Physiol Biochem. (2015) 35:246–58. 10.1159/000369692 25591767

[80] Han F Huo Y Huang CJ Chen CL Ye J. MicroRNA-30b promotes axon outgrowth of retinal ganglion cells by inhibiting Semaphorin3A expression. Brain Res. (2015) 1611:65–73. 10.1016/j.brainres.2015.03.014 25791621

[81] Hancock ML Preitner N Quan J Flanagan JG. MicroRNA-132 is enriched in developing axons, locally regulates Rasa1 mRNA, and promotes axon extension. J Neurosci. (2014) 34:66–78. 10.1523/JNEUROSCI.3371-13.2014 24381269 PMC3866495

[82] Jiao S Liu Y Yao Y Teng J. miR-124 promotes proliferation and neural differentiation of neural stem cells through targeting DACT1 and activating Wnt/β-catenin pathways. Mol Cell Biochem. (2018) 449:305–14. 10.1007/s11010-018-3367-z 29786763

[83] Xiao WZ Lu AQ Liu XW Li Z Zi Y Wang ZW. Role of miRNA-146 in proliferation and differentiation of mouse neural stem cells. Biosci Rep. (2015) 35:e00245. 10.1042/BSR20150088 26199426 PMC4613701

[84] Li Z Song Y He T Wen R Li Y Chen T. M2 microglial small extracellular vesicles reduce glial scar formation *via* the miR-124/STAT3 pathway after ischemic stroke in mice. Theranostics. (2021) 11:1232–48. 10.7150/thno.48761 33391532 PMC7738903

[85] Mirzaei H Momeni F Saadatpour L Sahebkar A Goodarzi M Masoudifar A. MicroRNA: relevance to stroke diagnosis, prognosis, and therapy. J Cell Physiol. (2018) 233:856–65. 10.1002/jcp.25787 28067403

[86] Huang L Wu ZB Zhuge Q Zheng W Shao B Wang B. Glial scar formation occurs in the human brain after ischemic stroke. Int J Med Sci. (2014) 11:344–8. 10.7150/ijms.8140 24578611 PMC3936028

[87] Fujita Y Yamashita T. Axon growth inhibition by RhoA/ROCK in the central nervous system. Front Neurosci. (2014) 8:338. 10.3389/fnins.2014.00338 25374504 PMC4205828

[88] Wang J Ni G Liu Y Han Y Jia L Wang Y. Tanshinone IIA Promotes axonal regeneration in rats with focal cerebral ischemia through the inhibition of nogo-A/NgR1/RhoA/ROCKII/MLC signaling. Drug Des Devel Ther. (2020) 14:2775–87 10.2147/DDDT.S253280 32764877 PMC7371607

[89] Griffin JW George R Lobato C Tyor WR Yan LC Glass JD. Macrophage responses and myelin clearance during Wallerian degeneration: relevance to immune-mediated demyelination. J Neuroimmunol. (1992) 40:153–65. 10.1016/0165-5728(92)90129-9 1430148

[90] George R Griffin JW. Delayed macrophage responses and myelin clearance during Wallerian degeneration in the central nervous system: the dorsal radiculotomy model. Exp Neurol. (1994) 129:225–36. 10.1006/exnr.1994.1164 7957737

[91] Ishii A Furusho M Bansal R. Mek/ERK1/2-MAPK and PI3K/Akt/mTOR signaling plays both independent and cooperative roles in Schwann cell differentiation, myelination and dysmyelination. Glia. (2021) 69:2429–46. 10.1002/glia.24049 34157170 PMC8373720

[92] Kuhn S Gritti L Crooks D Dombrowski Y. Oligodendrocytes in development, myelin generation and beyond. Cells. (2019) 8:1424. 10.3390/cells8111424 31726662 PMC6912544

[93] Yiu G He Z. Glial inhibition of CNS axon regeneration. Nat Rev Neurosci. (2006) 7:617–27. 10.1038/nrn1956 16858390 PMC2693386

[94] GrandPré T Nakamura F Vartanian T Strittmatter SM. Identification of the Nogo inhibitor of axon regeneration as a Reticulon protein. Nature. (2000) 403:439–44. 10.1038/35000226 10667797

[95] Orfila JE Dietz RM Rodgers KM Dingman A Patsos OP Cruz-Torres I. Experimental pediatric stroke shows age-specific recovery of cognition and role of hippocampal Nogo-A receptor signaling. J Cereb Blood Flow Metab. (2020) 40:588–99. 10.1177/0271678X19828581 30762478 PMC7026845

[96] Zhai Z Guo Y. Combination of constraint-induced movement therapy with fasudil amplifies neurogenesis after focal cerebral ischemia/reperfusion in rats. Int J Neurosci. (2022) 132:1254–60. 10.1080/00207454.2021.1881088 33527868

[97] Tsai SY Papadopoulos CM Schwab ME Kartje GL. Delayed anti-nogo-a therapy improves function after chronic stroke in adult rats. Stroke. (2011) 42:186–90. 10.1161/STROKEAHA.110.590083 21088244 PMC3806087

[98] Rust R Grönnert L Gantner C Enzler A Mulders G Weber RZ. Nogo-A targeted therapy promotes vascular repair and functional recovery following stroke. Proc Natl Acad Sci U S A. (2019) 116:14270–9. 10.1073/pnas.1905309116 31235580 PMC6628809

[99] Zhou D Cen K Liu W Liu F Liu R Sun Y. Xuesaitong exerts long-term neuroprotection for stroke recovery by inhibiting the ROCKII pathway, *in vitro* and *in vivo*. J Ethnopharmacol. (2021) 272:113943. 10.1016/j.jep.2021.113943 33617967

[100] Mi S Lee X Shao Z Thill G Ji B Relton J. LINGO-1 is a component of the Nogo-66 receptor/p75 signaling complex. Nat Neurosci. (2004) 7:221–8. 10.1038/nn1188 14966521

[101] Schwab ME. Functions of Nogo proteins and their receptors in the nervous system. Nat Rev Neurosci. (2010) 11:799–811. 10.1038/nrn2936 21045861

[102] Wahl AS Correa D Imobersteg S Maurer MA Kaiser J Augath MA. Targeting therapeutic antibodies to the CNS: a comparative study of intrathecal, intravenous, and subcutaneous anti-Nogo A antibody treatment after stroke in rats. Neurotherapeutics. (2020) 17:1153–9. 10.1007/s13311-020-00864-z 32378027 PMC7609675

[103] Wang KC Kim JA Sivasankaran R Segal R He Z. P75 interacts with the Nogo receptor as a co-receptor for Nogo, MAG and OMgp. Nature. (2002) 420:74–8. 10.1038/nature01176 12422217

[104] Wong ST Henley JR Kanning KC Huang KH Bothwell M Poo MM. p75(NTR) and Nogo receptor complex mediates repulsive signaling by myelin-associated glycoprotein. Nat Neurosci. (2002) 5:1302–8. 10.1038/nn975 12426574

[105] Sekine Y Algarate PT Cafferty W Strittmatter SM. Plexina2 and CRMP2 signaling complex is activated by Nogo-A-liganded Ngr1 to restrict corticospinal axon sprouting after trauma. J Neurosci. (2019) 39:3204–16. 10.1523/JNEUROSCI.2996-18.2019 30804090 PMC6788813

[106] Fournier AE GrandPre T Strittmatter SM. Identification of a receptor mediating Nogo-66 inhibition of axonal regeneration. Nature. (2001) 409:341–6. 10.1038/35053072 11201742

[107] Li L Deng B Wang S Zhong H Liu Z Jin W. Asynchronous therapy targeting Nogo-A enhances neurobehavioral recovery by reducing neuronal loss and promoting neurite outgrowth after cerebral ischemia in mice. J Drug Target. (2016) 24:13–23. 10.3109/1061186X.2015.1052070 26061295

[108] Gou X Wang Q Yang Q Xu L Xiong L. TAT-NEP1-40 as a novel therapeutic candidate for axonal regeneration and functional recovery after stroke. J Drug Target. (2011) 19:86–95. 10.3109/10611861003733961 20367026

[109] Atwal JK Pinkston-Gosse J Syken J Stawicki S Wu Y Shatz C. PirB is a functional receptor for myelin inhibitors of axonal regeneration. Science. (2008) 322:967–70. 10.1126/science.1161151 18988857

[110] Filbin MT. PirB, a second receptor for the myelin inhibitors of axonal regeneration Nogo66, MAG, and OMgp: implications for regeneration *in vivo*. Neuron. (2008) 60:740–2. 10.1016/j.neuron.2008.12.001 19081369

[111] Taylor J Chung KH Figueroa C Zurawski J Dickson HM Brace EJ. The scaffold protein POSH regulates axon outgrowth. Mol Biol Cell. (2008) 19:5181–92. 10.1091/mbc.e08-02-0231 18829867 PMC2592661

[112] Deng B Li L Gou X Xu H Zhao Z Wang Q. TAT-PEP enhanced neurobehavioral functional recovery by facilitating axonal regeneration and corticospinal tract projection after stroke. Mol Neurobiol. (2018) 55:652–67. 10.1007/s12035-016-0301-9 27987133

[113] Yang M Jian L Fan W Chen X Zou H Huang Y. Axon regeneration after optic nerve injury in rats can be improved *via* PirB knockdown in the retina. Cell Biosci. (2021) 11:158. 10.1186/s13578-021-00670-w 34380548 PMC8359350

[114] Li C Wen H Wang Q Zhang C Jiang L Dou Z. Exercise training inhibits the Nogo-A/NgR1/Rho-A signals in the cortical peri-infarct area in hypertensive stroke rats. Am J Phys Med Rehabil. (2015) 94:1083–94. 10.1097/PHM.0000000000000339 26135366

[115] Adelson JD Barreto GE Xu L Kim T Brott BK Ouyang YB. Neuroprotection from stroke in the absence of MHCI or PirB. Neuron. (2012) 73:1100–7. 10.1016/j.neuron.2012.01.020 22445338 PMC3314229

[116] Quarles RH. Myelin-associated glycoprotein (MAG): past, present and beyond. J Neurochem. (2007) 100:1431–48. 10.1111/j.1471-4159.2006.04319.x 17241126

[117] Kastin AJ Pan W. Targeting neurite growth inhibitors to induce CNS regeneration. Curr Pharm Des. (2005) 11:1247–53. 10.2174/1381612053507440 15853681

[118] Wang R Zhao H Li J Duan Y Fan Z Tao Z. Erythropoietin attenuates axonal injury after middle cerebral artery occlusion in mice. Neurol Res. (2017) 39:545–51. 10.1080/01616412.2017.1316904 28413924

[119] Habib AA Marton LS Allwardt B Gulcher JR Mikol DD Högnason T. Expression of the oligodendrocyte-myelin glycoprotein by neurons in the mouse central nervous system. J Neurochem. (1998) 70:1704–11. 10.1046/j.1471-4159.1998.70041704.x 9523589

[120] Wang KC Koprivica V Kim JA Sivasankaran R Guo Y Neve RL. Oligodendrocyte-myelin glycoprotein is a Nogo receptor ligand that inhibits neurite outgrowth. Nature. (2002) 417:941–4. 10.1038/nature00867 12068310

[121] Choudhury GR Ding S. Reactive astrocytes and therapeutic potential in focal ischemic stroke. Neurobiol Dis. (2016) 85:234–44. 10.1016/j.nbd.2015.05.003 25982835 PMC4644522

[122] Rolls A Shechter R Schwartz M. The bright side of the glial scar in CNS repair. Nat Rev Neurosci. (2009) 10:235–41. 10.1038/nrn2591 19229242

[123] Abeysinghe HC Phillips EL Chin-Cheng H Beart PM Roulston CL. Modulating astrocyte transition after stroke to promote brain rescue and functional recovery: emerging targets include rho kinase. Int J Mol Sci. (2016) 17:288. 10.3390/ijms17030288 26927079 PMC4813152

[124] Sofroniew MV. Molecular dissection of reactive astrogliosis and glial scar formation. Trends Neurosci. (2009) 32:638–47. 10.1016/j.tins.2009.08.002 19782411 PMC2787735

[125] Kim J Sajid MS Trakhtenberg EF. The extent of extra-axonal tissue damage determines the levels of CSPG upregulation and the success of experimental axon regeneration in the CNS. Sci Rep. (2018) 8:9839. 10.1038/s41598-018-28209-z 29959434 PMC6026156

[126] Tran AP Warren PM Silver J. Regulation of autophagy by inhibitory CSPG interactions with receptor PTPσ and its impact on plasticity and regeneration after spinal cord injury. Exp Neurol. (2020) 328:113276. 10.1016/j.expneurol.2020.113276 32145250 PMC7145755

[127] Kaprielian Z Runko E Imondi R. Axon guidance at the midline choice point. Dev Dyn. (2001) 221:154–81. 10.1002/dvdy.1143 11376484

[128] Flanagan JG Van Vactor D. Through the looking glass: axon guidance at the midline choice point. Cell. (1998) 92:429–32. 10.1016/S0092-8674(00)80935-6 9491883

[129] Raper J Mason C. Cellular strategies of axonal pathfinding. Cold Spring Harb Perspect Biol. (2010) 2:a001933. 10.1101/cshperspect.a001933 20591992 PMC2926747

[130] Van Battum EY Brignani S Pasterkamp RJ. Axon guidance proteins in neurological disorders. Lancet Neurol. (2015) 14:532–46. 10.1016/S1474-4422(14)70257-1 25769423

[131] Stoeckli ET. Understanding axon guidance: are we nearly there yet. Development. (2018) 145:dev151415. 10.1242/dev.151415 29759980

[132] Omotade OF Pollitt SL Zheng JQ. Actin-based growth cone motility and guidance. Mol Cell Neurosci. (2017) 84:4–10. 10.1016/j.mcn.2017.03.001 28268126 PMC5587356

[133] Zang Y Chaudhari K Bashaw GJ. New insights into the molecular mechanisms of axon guidance receptor regulation and signaling. Curr Top Dev Biol. (2021) 142:147–96. 10.1016/bs.ctdb.2020.11.008 33706917 PMC8456978

[134] Kolodkin AL Tessier-Lavigne M. Mechanisms and molecules of neuronal wiring: a primer. Cold Spring Harb Perspect Biol. (2011) 3:a001727. 10.1101/cshperspect.a001727 21123392 PMC3098670

[135] Kennedy TE. Cellular mechanisms of netrin function: long-range and short-range actions. Biochem Cell Biol. (2000) 78:569–75. 10.1139/o00-079 11103947

[136] Wang KH Brose K Arnott D Kidd T Goodman CS Henzel W. Biochemical purification of a mammalian slit protein as a positive regulator of sensory axon elongation and branching. Cell. (1999) 96:771–84. 10.1016/S0092-8674(00)80588-7 10102266

[137] Tong M Jun T Nie Y Hao J Fan D. The Role of the Slit/Robo signaling pathway. J Cancer. (2019) 10:2694–705. 10.7150/jca.31877 31258778 PMC6584916

[138] Ypsilanti AR Zagar Y Chédotal A. Moving away from the midline: new developments for Slit and Robo. Development. (2010) 137:1939–52. 10.1242/dev.044511 20501589

[139] Yeh ML Gonda Y Mommersteeg MT Barber M Ypsilanti AR Hanashima C. Robo1 modulates proliferation and neurogenesis in the developing neocortex. J Neurosci. (2014) 34:5717–31. 10.1523/JNEUROSCI.4256-13.2014 24741061 PMC3988420

[140] Kumanogoh A Kikutani H. Semaphorins and their receptors: novel features of neural guidance molecules. Proc Jpn Acad Ser B Phys Biol Sci. (2010) 86:611–20. 10.2183/pjab.86.611 20551597 PMC3081170

[141] Shirvan A Kimron M Holdengreber V Ziv I Ben-Shaul Y Melamed S. Anti-semaphorin 3A antibodies rescue retinal ganglion cells from cell death following optic nerve axotomy. J Biol Chem. (2002) 277:49799–807. 10.1074/jbc.M204793200 12376549

[142] Kania A Klein R. Mechanisms of ephrin-Eph signalling in development, physiology and disease. Nat Rev Mol Cell Biol. (2016) 17:240–56. 10.1038/nrm.2015.16 26790531

[143] Overman JJ Clarkson AN Wanner IB Overman WT Eckstein I Maguire JL. A role for ephrin-A5 in axonal sprouting, recovery, and activity-dependent plasticity after stroke. Proc Natl Acad Sci U S A. (2012) 109:E2230–9. 10.1073/pnas.1204386109 22837401 PMC3421211

[144] Drescher U Kremoser C Handwerker C Löschinger J Noda M Bonhoeffer F. *In vitro* guidance of retinal ganglion cell axons by RAGS, a 25 kDa tectal protein related to ligands for Eph receptor tyrosine kinases. Cell. (1995) 82:359–70. 10.1016/0092-8674(95)90425-5 7634326

[145] Hodge RG Ridley AJ. Regulating Rho GTPases and their regulators. Nat Rev Mol Cell Biol. (2016) 17:496–510. 10.1038/nrm.2016.67 27301673

[146] Mackay DJ Esch F Furthmayr H Hall A. Rho- and rac-dependent assembly of focal adhesion complexes and actin filaments in permeabilized fibroblasts: an essential role for ezrin/radixin/moesin proteins. J Cell Biol. (1997) 138:927–38. 10.1083/jcb.138.4.927 9265657 PMC2138043

[147] Boncoraglio GB Ranieri M Bersano A Parati EA Del Giovane C. Stem cell transplantation for ischemic stroke. Cochrane Database Syst Rev. (2019) 5:CD007231. 10.1002/14651858.CD007231.pub3 31055832 PMC6500737

[148] Savitz SI Dinsmore J Wu J Henderson GV Stieg P Caplan LR. Neurotransplantation of fetal porcine cells in patients with basal ganglia infarcts: a preliminary safety and feasibility study. Cerebrovasc Dis. (2005) 20:101–7. 10.1159/000086518 15976503

[149] Wang J Tian L Zhang Z Yuan B Zhang T Li X. Scalp-acupuncture for patients with hemiplegic paralysis of acute ischaemic stroke: a randomized controlled clinical trial. J Tradit Chin Med. (2020) 40:845–54. 10.19852/j.cnki.jtcm.2020.05.015 33000586

[150] Zhou LF. Effects of Electroacupuncture on the eXpression of BDNF, Sema3A, and NRP-1 in Rats With Focal Cerebral Infarction. MA thesis. Nanning: Guangxi Medical University (2019)

[151] Kim YR Kim HN Ahn SM Choi YH Shin HK Choi BT. Electroacupuncture promotes post-stroke functional recovery *via* enhancing endogenous neurogenesis in mouse focal cerebral ischemia. PLoS ONE. (2014) 9:e90000. 10.1371/journal.pone.0090000 24587178 PMC3933702

[152] Kim MW Chung YC Jung HC Park MS Han YM Chung YA. Electroacupuncture enhances motor recovery performance with brain-derived neurotrophic factor expression in rats with cerebral infarction. Acupunct Med. (2012) 30:222–6. 10.1136/acupmed-2011-010126 22729070

[153] Yi W Xu NG Wang GB. [Experimental study on effects of electro-acupuncture in improving synaptic plasticity in focal cerebral ischemia rats]. Zhongguo Zhong Xi Yi Jie He Za Zhi. (2006) 26:710–4. 16970094

[154] Xia WG Zheng CJ Zhang X Wang J. Effects of “nourishing liver and kidney” acupuncture therapy on expression of brain derived neurotrophic factor and synaptophysin after cerebral ischemia reperfusion in rats. J Huazhong Univ Sci Technolog Med Sci. (2017) 37:271–8. 10.1007/s11596-017-1727-7 28397041

[155] Zheng Y Qin Z Tsoi B Shen J Zhang ZJ. Electroacupuncture on trigeminal nerve-innervated acupoints ameliorates poststroke cognitive impairment in rats with middle cerebral artery occlusion: involvement of neuroprotection and synaptic plasticity. Neural Plast. (2020) 2020:8818328. 10.1155/2020/8818328 32963517 PMC7492933

[156] Zhao Y Mao X Wang H Gan L Zhang S Gong P. The influence of electronic acupuncture at a specific frequency in facilitating the passage of NGF through the blood-brain barrier and its effect on learning and memory in MCAO/R rats. J Integr Neurosci. (2022) 21:79. 10.31083/j.jin2103079 35633160

[157] Zhang XB Wang Y Wang Z Kong H Wang J Jiang L. Effect of nape cluster acupuncture on BDNF, NGF and neurobehaviors in rats with post-ischemic stroke sequelae. Shanghai J Acupunct Moxibustion. (2014) 33:181–4. 10.13460/j.issn.1005-0957.2014.02.0181

[158] Tao J Zheng Y Liu W Yang S Huang J Xue X. Electro-acupuncture at LI11 and ST36 acupoints exerts neuroprotective effects *via* reactive astrocyte proliferation after ischemia and reperfusion injury in rats. Brain Res Bull. (2016) 120:14–24. 10.1016/j.brainresbull.2015.10.011 26524137

[159] Gu WJ Bao CR Xie HY Chen B Lin WQ Wu Y. [Effect of electroacupuncture invention on activities of PGC-1α/Irisin (FNDC5)/BDNF signaling in cerebral cortex, hippocampus and muscles in focal cerebral ischemic/reperfusion injury rats]. Zhen Ci Yan Jiu. (2022) 47:428–34. 10.13702/j.1000-0607.20210495 35616417

[160] Chen A Lin Z Lan L Xie G Huang J Lin J. Electroacupuncture at the Quchi and Zusanli acupoints exerts neuroprotective role in cerebral ischemia-reperfusion injured rats *via* activation of the PI3K/Akt pathway. Int J Mol Med. (2012) 30:791–6. 10.3892/ijmm.2012.1074 22842715

[161] Xue X You Y Tao J Ye X Huang J Yang S. Electro-acupuncture at points of Zusanli and Quchi exerts anti-apoptotic effect through the modulation of PI3K/Akt signaling pathway. Neurosci Lett. (2014) 558:14–9. 10.1016/j.neulet.2013.10.029 24157854

[162] Yang ZJ Shen DH Guo X Sun FY. Electroacupuncture enhances striatal neurogenesis in adult rat brains after a transient cerebral middle artery occlusion. Acupunct Electrother Res. (2005) 30:185–99. 10.3727/036012905815901244 16617687

[163] Tao J Chen B Gao Y Yang S Huang J Jiang X. Electroacupuncture enhances hippocampal NSCs proliferation in cerebral ischemia-reperfusion injured rats *via* activation of notch signaling pathway. Int J Neurosci. (2014) 124:204–12. 10.3109/00207454.2013.840781 24004240

[164] Chen B Tao J Lin Y Lin R Liu W Chen L. Electro-acupuncture exerts beneficial effects against cerebral ischemia and promotes the proliferation of neural progenitor cells in the cortical peri-infarct area through the Wnt/β-catenin signaling pathway. Int J Mol Med. (2015) 36:1215–22. 10.3892/ijmm.2015.2334 26329606 PMC4601740

[165] Zhang S Jin T Wang L Liu W Zhang Y Zheng Y. Electro-acupuncture promotes the differentiation of endogenous neural stem cells *via* exosomal microRNA 146b after ischemic stroke. Front Cell Neurosci. (2020) 14:223. 10.3389/fncel.2020.00223 32792909 PMC7385414

[166] Zhao X Bai F Zhang E Zhou D Jiang T Zhou H. Electroacupuncture improves neurobehavioral function through targeting of SOX2-mediated axonal regeneration by microRNA-132 after ischemic stroke. Front Mol Neurosci. (2018) 11:471. 10.3389/fnmol.2018.00471 30618618 PMC6306468

[167] Xu SY Zeng CL Ni SM Peng YJ. The angiogenesis effects of electro-acupuncture treatment *via* exosomal miR-210 in cerebral ischemia-reperfusion rats. Curr Neurovasc Res. (2022) 19:61–72. 10.2174/1567202619666220321115412 35319370

[168] Tan F Chen J Liang YG Li YP Wang XW Meng D. [Effect of electric acupuncture on the expression of NgR in the cerebral cortex, the medulla oblongata, and the spinal cord of hypertensive rats after cerebral infarction]. Zhongguo Zhong Xi Yi Jie He Za Zhi. (2014) 34:334–41. 24758087

[169] Xie ZR Tang YN Pan J Cap Y Lou B. Effects of electroacupuncture stimulation of acupoints of pericardium meridian on Nogo-A and NgR1 expression in cerebral ischemia rats. Chin J Inf Tradit Chin Med. (2020) 27:59–63. 10.19879/i.cnki.1005-5304.202004131

[170] Chen S Wang H Xu H Zhang Y Sun H. Electroacupuncture promotes axonal regrowth by attenuating the myelin-associated inhibitors-induced RhoA/ROCK pathway in cerebral ischemia/reperfusion rats. Brain Res. (2020) 1748:147075. 10.1016/j.brainres.2020.147075 32853644

[171] Huang S Huang D Zhao J Chen L. Electroacupuncture promotes axonal regeneration in rats with focal cerebral ischemia through the downregulation of Nogo-A/NgR/RhoA/ROCK signaling. Exp Ther Med. (2017) 14:905–12. 10.3892/etm.2017.4621 28810542 PMC5526169

[172] Xiao LY Wang XR Yang Y Yang JW Cao Y Ma SM. Applications of acupuncture therapy in modulating plasticity of central nervous system. Neuromodulation. (2018) 21:762–76. 10.1111/ner.12724 29111577

